# Universal energy-space localization and stable quantum phases against time-dependent perturbations

**DOI:** 10.1038/s41467-026-75465-z

**Published:** 2026-07-14

**Authors:** Hongye Yu, Tzu-Chieh Wei

**Affiliations:** 1 C. N. Yang Institute for Theoretical Physics, State University of New York at Stony Brook, New York, NY USA; 2 Department of Physics and Astronomy, State University of New York at Stony Brook, Stony Brook, New York, NY USA

**Keywords:** Quantum information, Quantum mechanics, Information theory and computation, Theoretical physics, Thermodynamics

## Abstract

Stability against perturbations is a defining property of quantum many-body phases of matter. However, most rigorous stabilities are only established for static perturbations; whether any system can remain stable against generic time-dependent perturbations is largely elusive. Here, we identify a universal phenomenon, where the evolving state driven by time-dependent *q*-local Hamiltonians can be exponentially localized in an energy window of instantaneous spectrum, and prove its survival under generic time-dependent perturbations. Applying such energy-space localization to classical and quantum LDPC codes whose codewords are separated by extensive energy barriers, we show that the system remains localized near the original codeword for an exponentially long time under generic time-dependent perturbations. For classical optimization problems with clustered solution spaces, the stability becomes an obstacle for quantum Hamiltonian-based algorithms to escape local minima. Our work provides a new lens for analyzing quantum non-equilibrium dynamics and tools for establishing stability and designing quantum algorithms.

## Abstract (web summary)

Stability under generic time dependent perturbations remains poorly understood in quantum systems. Here, the authors identify a universal energy space localization property of driven q local Hamiltonians and show its consequences for robust dynamics in LDPC codes and hard optimization problems.

## Introduction

Over the past few decades, many new phases^1–5^ in condensed matter physics have been identified and discovered. Stability against perturbations is often both a requirement and a signal for defining a new phase of matter. Many of these phases, such as topologically ordered phases^2,6,7^, many-body localization^3,8^, prethermalization^5,9–11^, and some open quantum systems^12–14^, have been proven to be stable against certain types of perturbations. Some of these stable phases have also been experimentally realized^15–20^, but most theoretical proofs of their stability are limited to static perturbations, whereas most perturbations in reality are time-dependent. However, whether these phases are stable under time-dependent perturbations is unknown, and rigorous bounds and mathematical tools for analyzing generic time-dependent perturbations are lacking in the literature. For time-dependent perturbations, most existing methods^21–23^ give bounds in power series of evolution time *t*, which will eventually diverge with increasing *t* and thus can only control errors within short-time evolutions. These obstacles correspond to uncontrollable effects of generic time-dependent perturbations, such as high excitations and large heat absorption, which can drive the states globally away from their initial configurations. Thus, in general, we expect that many of those stable phases will no longer be robust against generic time-dependent perturbations under long-time driving.

Therefore, it is fundamentally important to ask: Are there any systems that do possess provable stability against generic time-dependent perturbations? We tackle this problem from a different perspective from many previous attempts: Instead of trying to prove stability for specific models, we first identify which property can survive under generic time-dependent perturbations and then use it as a guide to search for corresponding models. In this work, we identify one property called energy-space localization that survives under generic time-dependent perturbations and can be used as a guide to search for dynamically stable models. For an initial eigenstate that evolves under a generic time-dependent Hamiltonian *H*(*t*), it will become a superposition of instantaneous eigenstates and spread in the instantaneous energy space. If *H*(*t*) is *q*-local (not necessarily geometrically local) and has a bounded local norm, we can rigorously prove that the spreading is exponentially localized in an energy window near the initial energy, with a width roughly equal to the total variation of *H*(*t*), which is consistent with intuitions from classical mechanics. In addition, all the bounds we obtain are invariant under monotonic rescaling of *t*. This indicates that the far region of the spectrum remains inaccessible for all times to quantum evolution *H*(*t*) with small total variation, regardless of how rapidly or slowly *H*(*t*) varies.

Since energy-space localization will be shown to hold ubiquitously for physical *q*-local Hamiltonians *H*(*t*), itself alone may not necessarily yield additional non-trivial properties of the system. Indeed, according to the eigenstate thermalization hypothesis (ETH)^24–28^, a state localized at one single energy level of a generic Hamiltonian can behave like a thermal ensemble. Thus, localization in an energy window alone does not prevent states from thermalization or ergodicity in generic systems. On the other hand, systems that can break ergodicity are highly nontrivial, especially when such breaking persists under generic perturbations. This stability is indeed the property of some exotic phenomena, such as MBL^3^ and pretheramlization^10,29^. However, these existing models and theoretical proofs do not apply to generic time-dependent perturbations, and it is likely that they are no longer stable in this scenario. As we will prove that energy-space localization can survive under generic time-dependent perturbations for an arbitrarily long time, could it uncover new stability? How could stability emerge beyond energy space?

To search for a stable phase via energy-space localization, we need to relate the closeness in the energy space to that in the configuration space. This property indeed exists in certain spin glass models, which can be constructed from error-correcting codes^30–33^ and hard classical optimization problems^34^, where the low-energy eigenspace is well separated into distant clusters, each of which contains eigenstates that are close. In these cases, eigenstates require overcoming a significant energy barrier to reach other clusters. Using the energy-space localization that we shall derive, we can prove that the localization time in each cluster is exponentially long for linear barriers and superpolynomially long for sublinearly increasing barriers, provided the total variation of the time-dependent perturbation is smaller than half of the energy barrier. Such localization is a hallmark of ergodicity breaking, and is stronger than the usual context of ergodicity defined in static Hamiltonians.

Such localization is useful for quantum error-correcting codes, especially when used as fault-tolerant quantum memory^35–38^. A central question in this context is whether the error-correcting codes can protect quantum information under generic perturbations for a long time. Although realistic noises are generally time-dependent, rigorously proving stability under generic time-dependent perturbations was considered theoretically intractable, as there are rarely theoretical tools that can bound unitary dynamics of *H*(*t*) for long-time evolutions. Even for static perturbations, proofs for dynamical stability of quantum codes mostly rely on ground-state (or zero-temperature) stability^39–41^, which no longer works if the initial state already contains small correctable errors. Can erroneous initial states of certain qLDPC codes remain correctable after a long-time noisy evolution? Our results give an affirmative answer in the most general time-dependent setting: If the code exhibits linear energy barriers, errors induced by weak time-dependent perturbations remain correctable if the total variation imparted by the latter is small.

Our results can also be directly reduced to static cases by taking the time duration of time-dependent perturbations to the infinitesimal limit in a quench dynamics. This allows us to prove that the perturbed eigenstates are universally localized inside the energy-space of the unperturbed spectrum. One of its special cases is when the unperturbed Hamiltonian is mutually commuting. For such Hamiltonians, their energy-space localization was originally obtained in ref. ^33^, and was used as a stepping stone to prove the infinite-time dynamical localization of cLDPC codes via proving the localization of perturbed eigenstates. However, it remains unknown whether qLDPC codes can have eigenstate localization; thus, proving dynamical localization of qLDPC codes from static eigenstate localization is still not feasible. In this work, we bypass this obstacle by directly proving the dynamical localization of qLDPC codes in time-dependent cases, which naturally include static perturbations and yield an exponentially long localization time regardless of whether the systems exhibit eigenstate localization. The static reduction can also reproduce many existing stability results^14,33^ of spin glasses against static *q*-local perturbations and generalize them to quasi-*q*-local perturbations.

On the other hand, localization can also pose an obstacle to Hamiltonian-based quantum algorithms aiming to solve difficult classical optimization problems^34^ whose near-optimal solution space is clustered. The hardness from their clustered solutions was previously leveraged to rule out logarithmic-time quantum algorithms^42^. Although it is believed that such NP-hard problems remain hard within polynomial-time quantum algorithms, no rigorous results are known to rule out algorithms longer than linear time. In this work, we will show that for Hamiltonian-based quantum algorithms, the system will become trapped in local minima for an exponentially long time due to the energy barriers if the algorithmic Hamiltonian’s total variation is insufficient.

Finally, we will discuss how to experimentally detect the results obtained in this work. For energy-space localization itself, its exponentially small tail bounds are hard to directly measure. Instead, we can probe the unusual localization behavior of systems with extensive energy barriers to confirm our theory in potential experimental platforms.

## Results

### Energy-space localization

In this section, we mainly work with a generic *q*-local time-dependent Hamiltonian *H*(*t*) defined on an *n*-site lattice. To define a *q*-local Hamiltonian here, we require that *H*(*t*) admits a partition *H*(*t*) = ∑_*A*_*h*_*A*_(*t*) into *q*-local terms, where each *h*_*A*_(*t*) is supported in a region *A* that contains at most *q* sites. Here, we do not have any geometric constraints on the interacting region *A*, which can be spatially nonlocal. For example, the Ising model is 2-local and also geometrically local. All-to-all 2-body couplings are 2-local but not geometrically local. The Kitaev toric code^43^ is 4-local and geometrically local.

**Fig. 1:**
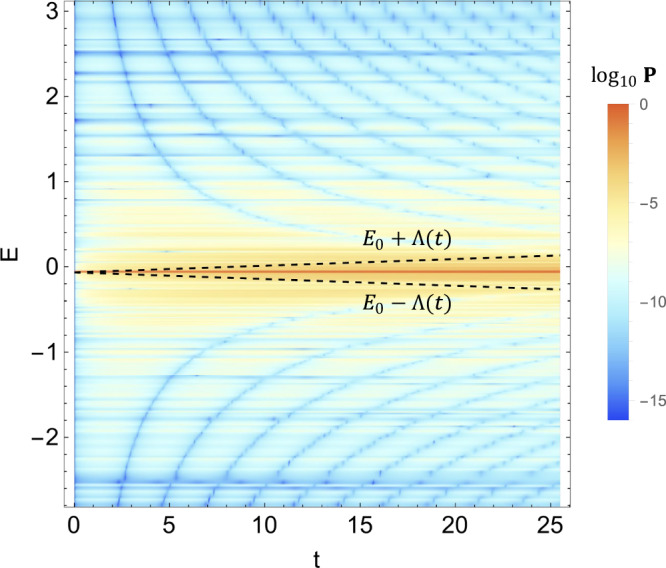
An illustrative example of the energy-space localization. *Λ*(*t*) denotes the total variation of *H*(*t*) from 0 to *t*. The results are from simulating an 8-qubit *H*(*t*) consisting of random 2-local Pauli operators with all-to-all couplings, where the initial state is chosen to be one eigenstate of *H*(0) with energy *E*_0_. The quantity **P** for *j*-th eigenstates of the instantaneous Hamiltonian *H*(*t*) is its probability in expanding the evolving state $\left\vert \psi (t)\right\rangle$ in the instantaneous spectrum.

Suppose a state $\left\vert \psi (t)\right\rangle$ is initialized at one eigenstate of *H*(0) at *t* = 0, which can also be viewed as a *δ*-function distribution of eigenstates in energy space. Let the state evolve under *H*(*t*); then $\left\vert \psi (t)\right\rangle$ will become a superposition of instantaneous eigenstates as long as *H*(*t*) at different times does not commute with one another. Thus the evolving state’s distribution in the energy space spreads. Our main results are on the localization of such spreading: The distribution is exponentially localized in a classically allowed energy window (see Fig. 1) for a generic physical *H*(*t*), and the width of the energy window is determined by the total variation of *H*(*t*) during the evolution. We will give formal statements on this argument shortly.

Before we proceed, we first introduce the requirements of a physical *q*-local *H*(*t*) in our context. Firstly, we require that the local norm ∥*H*(*t*)∥_loc_ is bounded. Here, the local norm defined by $\parallel H{\parallel }_{{{{\rm{loc}}}}}\equiv {{\sup }_{i}}{\sum }_{A,A\ni i}\parallel {h}_{A}\parallel$ in the local decomposition, which roughly measures the largest local strength of the Hamiltonian acting on a site. We also define the global termwise norm ∥*H*(*t*)∥_X_ = ∑_*A*_∥*h*_*A*_(*t*)∥ for convenience, which measures an absolute strength of the total Hamiltonian. We summarize the required conditions for *H*(*t*) as follows.

#### Case 1

(General) *H*(*t*) is *q*-local and ∥*H*(*t*)∥_loc_≤*M*, with $\int_{0}^{T}\parallel {{{\rm{d}}}}H{\parallel }_{{{{\rm{X}}}}}\equiv \int_{0}^{T}\parallel H^{\prime} (t){\parallel }_{{{{\rm{X}}}}}{{{\rm{d}}}}t\le \lambda n$,

where *λ* and *M* are constants independent of *n* and we call $\int_{0}^{T}\parallel {{{\rm{d}}}}H{\parallel }_{{{{\rm{X}}}}}$*t*otal variation of *H*(*t*) from *t* = 0 to *T* (see the formal definitions in Appendix A of the Supplementary Information).

We list below other conditions that are not included in Case 1, but will be useful as they have correspondence in the static scenarios.

#### Case 2

*H*(*t*) is *q*-local and can be written as *H*(*t*) = *H*_*C*_(*t*) + *V*(*t*) with $[V(t),H{^\prime} (t)]=0$ and ∥*H*_*C*_(*t*)∥_loc_≤*M*, with $\int_{0}^{T}\parallel {{{\rm{d}}}}H{\parallel }_{{{{\rm{X}}}}}\le \lambda n$.

Here, we do not require *H*_*C*_(*t*) to be mutually commuting, which is different from Cases 3 and 4.

#### Case 3

$H^{\prime} (t)$ is *q*-local. *H*(*t*) can be partitioned into *H*(*t*) = *H*_*C*_(*t*) + *V*(*t*) with *H*_*C*_(*t*) mutually commuting and ∥*H*_*C*_(*t*)∥_loc_≤*M*, and $[V(t),H^{\prime} (t)]=0$, with $\int_{0}^{T}\parallel {{{\rm{d}}}}H{\parallel }_{{{{\rm{X}}}}}\le \lambda n$.

#### Case 4

$H^{\prime} (t)$ is quasi-*q*-local. *H*(*t*) can be partitioned into *H*(*t*) = *H*_*C*_(*t*) + *V*(*t*) with *H*_*C*_(*t*) mutually commuting and ∥*H*_*C*_(*t*)∥_loc_≤*M*, and $[V(t),H^{\prime} (t)]=0$, with $\int_{0}^{T}\parallel {{{\rm{d}}}}H{\parallel }_{q-{{{\rm{X}}}}}\le \lambda n$,

where we define a Hamiltonian to be quasi-*q*-local if the strength of its *k*-local terms decays with *e*^−*k*/*q*^, and the termwise *q*-norm ∥ ⋅ ∥_*q*−X_ for quasi-*q*-local Hamiltonian as the total norm with weights *e*^*k*/*q*^ for each *k*-local term. Although intrinsic quasi-*q*-local interactions have not yet been found in reality, they arise naturally in many perturbative analyses and effective models ^9,10,29^.

We refer the reader to Table 1 and Appendix A in the Supplementary Information for the explicit definitions and properties of different norms and total variations. With these preparations, we can present our main results, which we call energy-space localization.

**Table 1:** Summary on definitions of different norms used in this work for a given decomposition of a Hamiltonian *H* = ∑_*A*_*h*_*A*_

Norms	Definitions
∥*H*∥	${{\sup }_{\left\vert \psi \right\rangle \ne 0}}\frac{\parallel H\left\vert \psi \right\rangle \parallel }{\parallel \left\vert \psi \right\rangle \parallel }$
∥*H*∥_loc_	${{\sup }_{i}}{\sum }_{A,A\ni i}\parallel {h}_{A}\parallel$
∥*H*∥_X_	∑_*A*_∥*h*_*A*_∥
∥*H*∥_*q*−X_	(*q* + 1)∑_*A*_*e*^∣*A*∣/*q*^∥*h*_*A*_∥

In the definition of ∥*H*∥_*q*−X_, ∣*A*∣ denotes the size of the region *A*, and *q* can be chosen from any positive real numbers. We refer the reader to Appendix A of the Supplementary Information for detailed definitions and properties of different norms. We define *H* as quasi-*q*_⋆_-local if one can find a *q*_⋆_ (usually the minimum of all possible *q*) such that $\frac{1}{n}\parallel H{\parallel }_{{q}_{\star }{{{\rm{-X}}}}}$ can be bounded by an *O*(1) constant.

#### Theorem 1

(Energy-space localization, informal)

We set an initial state $\left\vert \psi (0)\right\rangle$ as an eigenstate of *H*(0) with energy *E*_0_. If we let the state evolve according to *H*(*t*) from *t* = 0 to *T*, then the state at any *t* is exponentially localized in the energy window ${{{{\mathcal{E}}}}}_{0}(d)\equiv [{E}_{0}-dn,{E}_{0}+dn]$ in the instantaneous spectrum of *H*(*t*), where *d* can be any *Θ*(1) constant satisfying *d* > *λ*.

(I) For *H*(*t*) defined in Case 1, 2 and 4, the leakage *ϵ*(*d*) (defined as the norm of outside eigenstates) to instantaneous eigenstates with energy differences ∣*E*(*t*) − *E*_0_∣≥*dn* is bounded by

1 $${\epsilon }_{\lambda,\Delta }^{(1)}(d)\equiv \exp \left(-n\frac{\lambda }{\Delta }\left(\frac{d}{\lambda }-1-\ln \frac{d}{\lambda }+o(1)\right)\right),$$

where *Δ* = *Δ*_*q*_ ≡ 2*qM*.

(II) If *H*(*t*) satisfies Case 3, the bound can be improved to

2 $${\epsilon }_{\lambda,\Delta }^{(2)}(d)\equiv \exp \left(-n\frac{\lambda }{\Delta }\left(\frac{d}{\lambda }\ln \frac{d}{\lambda }-(\frac{d}{\lambda }-1)\right)\right),$$

where *Δ* = *Δ*_*q*_.

We present the formal statements and rigorous proofs in Appendix B in the Supplementary Information and provide more extensions and corollaries in Methods. Given the exponential decay of the bound for any *d* > *λ*, we can interpret from the theorem that eigenstates outside ${{{{\mathcal{E}}}}}_{0}(\lambda )$ have almost no contribution to the final state $\left\vert \psi (T)\right\rangle$ in the thermodynamic limit (see Fig. 1). On the other hand, it is unlikely to give universal bounds inside ${{{{\mathcal{E}}}}}_{0}(\lambda )$, as one can always choose $V(t)=E^{\prime} -{E}_{0}$ to precisely pinpoint the final state at any energy $E^{\prime} \in {{{{\mathcal{E}}}}}_{0}(\lambda )$.

The energy window ${{{{\mathcal{E}}}}}_{0}(\lambda )$ is consistent with classical intuitions: The energy change of a classical $\tilde{H}(t)$ is exactly $\int_{0}^{T}\tilde{H^{\prime}} (t){{{\rm{d}}}}t$ and can be bounded by $\int_{0}^{T}| \tilde{H^{\prime}} (t)| {{{\rm{d}}}}t\le \lambda n$. As $\tilde{H}(t)$ is classical here, the norm is simply the absolute value. Thus, the energy window ${{{{\mathcal{E}}}}}_{0}(\lambda )$ corresponds to the classically reachable region from the driving *H*(*t*). At the thermodynamic limit (*n* → *∞*), the energy-space localization of quantum *H*(*t*) is naturally reduced to classical mechanics: The contributions from the classically forbidden region (outside ${{{{\mathcal{E}}}}}_{0}(\lambda )$) in the final state vanish exponentially.

As the leakage bound is controlled by the total variation of *H*(*t*), it is invariant under any monotonic rescaling of any part of the evolution time. Thus, it is straightforward to reduce Theorem 1 to static perturbations *H* = *H*_0_ + *V*_0_, which is sketched in Methods. In this scenario, a common task is to study the properties of the eigenstates of *H* in the *u*nperturbed eigenbasis of *H*_0_, which is often easier to handle. We consider the following general static case:

#### Case 2′

(General) *H*_0_ is *q*-local and ∥*H*_0_∥_loc_≤*M*, with *q*-local ∥*V*_0_∥_X_≤*λn*.

The Case ${2}^{{\prime} }$ can be obtained by setting *H*_*C*_(*t*) = *H*_0_, *V*(*t*) = (1 − *t*/*T*)*V*_0_ and let *T* → 0^+^ in Case 2. Other time-dependent cases can also be similarly translated into static cases as follows:

#### Case 3′

*H*_0_ is mutually commuting and ∥*H*_0_∥_loc_≤*M*, with *q*-local ∥*V*_0_∥_X_≤*λn*.

#### Case 4 ′

*H*_0_ is mutually commuting and ∥*H*_0_∥_loc_≤*M*, with quasi-*q*-local ∥*V*_0_∥_*q*−X_≤*λn*.

These are the counterparts of Cases 3 and 4 in time-dependent cases. We summarize the energy-space localization for the static cases in the theorem below.

#### Theorem 2

(Energy-space localization, static cases)

Let $\left\vert \psi \right\rangle$ be an eigenstate of the combined Hamiltonian *H* = *H*_0_ + *V*_0_ with energy $E^{\prime}$. If *H*_0_ and *V*_0_ satisfy any of the above cases, then in the eigenbasis of *H*_0_, $\left\vert \psi \right\rangle$ is exponentially localized in the energy window $[E^{\prime} -dn,E^{\prime}+dn]$, where *d* can be any *Θ*(1) constant satisfying *d* > *λ*, with leakage bounded by ${\epsilon }_{\lambda,{\Delta }_{q}}^{(1)}(d)$ for Case ${2}^{{\prime} }$ and Case ${4}^{{\prime} }$, and ${\epsilon }_{\lambda,{\Delta }_{q}}^{(2)}(d)$ for mutually commuting *H*_0_ (Case ${3}^{{\prime} }$).

We will provide a simple proof for this theorem in Methods by taking the evolution time in Theorem 1 to an infinitesimal limit.

We remark that although the energy-space localization is consistent with classical intuitions, its universality and the explicit tail bounds have not been obtained before, even in generic static cases. Proposition 2 in the Supplementary Material of Ref. ^33^ obtained a similar bound to ${\epsilon }_{\lambda,{\Delta }_{q}}^{(2)}$ for mutually commuting *H*_0_ by rewriting the Hamiltonian in a tri-diagonal form, and the result can be regarded as a special case (Case ${3}^{{\prime} }$) in our Theorem 2. However, it is not clear how to generalize the tri-diagonal method to non-commuting *H*_0_ or quasi-*q*-local perturbations in Case ${2}^{{\prime} }$ and Case ${4}^{{\prime} }$, whereas our results bypass this obstacle by reducing from time-dependent cases in Theorem 1.

Given the universality of energy-space localization, one wonders whether there are specific scenarios in which it can be used to infer stronger stability. Surprisingly, when combined with a clustered energy landscape where the closeness in energy can infer closeness in configuration space, our energy-space localization can become a powerful tool to prove numerous stabilities for such systems. This clustering property has been proven in many spin glass models^44–47^, which we define formally in our context as follows:

#### Definition 1

(Clustering property)

A Hamiltonian *H* has the clustering property in an energy window [*E*_1_, *E*_2_] if all eigenstates in this energy window can be divided into clusters {*w*_*j*_} such that: For any two eigenstates $\left\vert \phi \right\rangle$ from *w*_*j*_ and $\left\vert \phi ^{\prime} \right\rangle$ from ${w}_{j^{\prime} }$, the distance satisfies ${{{\bf{D}}}}(\left\vert \phi \right\rangle,\left\vert \phi^ {\prime} \right\rangle )\le {\nu }_{1}$ if they are within the same cluster ${w}_{j}={w}_{j^{\prime} }$ and ${{{\bf{D}}}}(\left\vert \phi \right\rangle,\left\vert \phi {\prime} \right\rangle )\ge {\nu }_{2}$ if they come from different clusters ${w}_{j}\ne {w}_{j^{\prime} }$.

Here, we define the distance **D** as follows: ${{{\bf{D}}}}(\left\vert \phi \right\rangle,\left\vert \phi ^{\prime} \right\rangle )=D$ if *k*-local operators *V* cannot produce a nonzero $\langle \phi | V| \phi^ {\prime} \rangle$ with *k* < *D* but can produce with *k* = *D*. For distances between *Z*-basis states in qubit systems, the above definition is equivalent to the Hamming distance of classical bits.

**Fig. 2:**
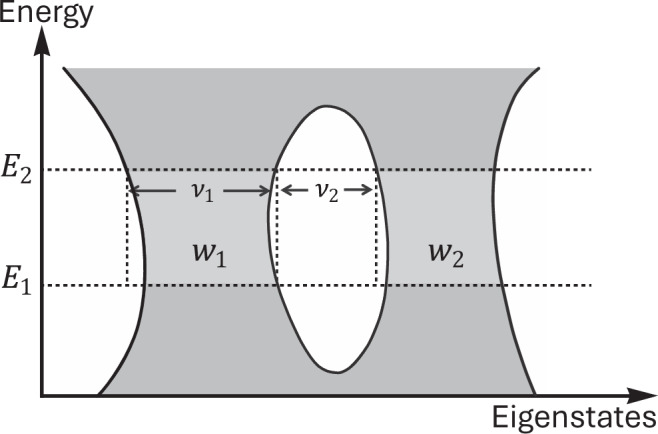
An illustrative example of the clustering property in the energy window [*E*_1_, *E*_2_] with maximum cluster diameter *ν*_1_ and minimum inter-cluster distances *ν*_2_, where the gray area denotes all the eigenstates and lighter gray areas denote two separated eigenstate clusters *w*_1_ and *w*_2_. We compress the high-dimensional eigenstates to one dimension for visual convenience, and the distance along the x-axis roughly represents the distance **D** between two eigenstates.

In this definition, the interval [*E*_1_, *E*_2_] acts as an energy barrier for different clusters (see Fig. 2). We require *ν*_2_ to be sufficiently large compared to the locality of the perturbations. When considering *q*-local perturbations, *ν*_2_ > *q* is usually sufficient. For quasi-*q*-local perturbations, we require *ν*_2_ ~ *Θ*(*n*). Although we mainly focus on models with *ν*_2_ ~ *Θ*(*n*) in this work, the results can easily extend to other scalings of *ν*_2_. Although we have no restriction on *ν*_1_, for localization to be well-defined, typically *ν*_1_ needs to be small compared to *n*, e.g., *ν*_1_ < *δn* with *δ* ≪ 1. We will show below that if the width *B* ≡ *E*_2_ − *E*_1_ of the energy window is linear with *n*, the states within each cluster exhibit stability against various types of perturbations, ranging from static to time-dependent, strictly *q*-local to quasi-*q*-local, and from close to open systems (see Table 2).

**Table 2:** Summary on the stabilities of LDPC codes with linear soundness discussed in this work

	Dynamical localization	Eigenstate localization	Slow mixing of Gibbs sampler
Perturbation type	(localization time)	(leakage)	(mixing time)
Static strictly *q*-local	*e*^*Ω*(*n*)^ [II.1], c: *∞*^33^	c: *e*^−*Ω*(*n*)^^33^	*e*^*Ω*(*n*)^^14,56^
Static quasi-*q*_⋆_-local	*e*^*Ω*(*n*)^ [II.1], c: *∞* [IV.2]	c: *e*^−*Ω*(*n*)^[IV.1]	*e*^*Ω*(*n*)^ [II.2]
Time-dependent (quasi-)*q*-local	*e*^*Ω*(*n*)^ [II.1]	-	-

The notation c denotes that the bound only works for cLDPC codes. Otherwise, the bound works for both cLDPC and qLDPC codes. More detailed bounds for parameters can be found in Table S1 in the Supplementary Information.

This property can appear in certain error-correcting codes and hard classical optimization problems. The stability is useful for quantum error-correcting codes, where the trapped states correspond to errors that can be easily detected and corrected to the original codeword. On the other hand, such stability can also pose an algorithmic barrier for optimization problems whose solution space exhibits the clustering property, where the evolution under weak driving cannot drive the trapped states out of local minima.

### Localization as stability in LDPC codes

LDPC codes are widely used error-correcting codes in both classical and quantum computing. The CSS-type quantum LDPC (qLDPC) codes can be written in the following Hamiltonian form

3 $${H}_{C}=-\left({\sum}_{{c}^{X}}{\prod}_{i\in {c}^{X}}{X}_{i}+{\sum}_{{c}^{Z}}{\prod}_{i\in {c}^{Z}}{Z}_{i}\right),$$

where *c*^*X*^ and *c*^*Z*^ are parity checks for *X* and *Z* operators, respectively, and they are mutually commuting. In addition, the low-density property requires that the number of checks involving each site is bounded by an *O*(1) constant *p*_*C*_, so that we can set *M* = *p*_*C*_ in ∥*H*_*C*_∥_loc_≤*M*. The classical LDPC (cLDPC) codes can be regarded as a special case, whose *H*_*C*_ contains only *Z* checks.

The different ground states $\left\vert {{{\rm{w}}}}\right\rangle$ of the LDPC codes, usually called codewords, are degenerate and well separated in terms of distance **D**, so they can robustly encode information. When errors occur, the energy of the erroneous state increases. All excited eigenstates ${\left\vert {{{\rm{v}}}}\right\rangle }_{{{{\rm{w}}}}}$ for qLDPC codes can be defined by excitations from a codeword $\left\vert {{{\rm{w}}}}\right\rangle$ as

4 $${\left\vert {{{\rm{v}}}}\right\rangle }_{{{{\rm{w}}}}}\equiv {\prod}_{i,{v}_{i}^{X}=1}{X}_{i}{\prod}_{j,{v}_{j}^{Z}=1}{Z}_{j}\left\vert {{{\rm{w}}}}\right\rangle,$$

where ${{{\rm{v}}}}\equiv \{{v}_{1}^{X},..{v}_{n}^{X},{v}_{1}^{Z},..{v}_{n}^{Z}\}$ is a length-2*n* bit string denoting local Pauli errors. We can also define ${\left\vert {{{\rm{v}}}}\right\rangle }_{{{{\rm{w}}}}}\equiv {\prod }_{j,{v}_{j}^{X}=1}{X}_{j}\left\vert {{{\rm{w}}}}\right\rangle$ with ${{{\rm{v}}}}\equiv \{{v}_{1}^{X},..{v}_{n}^{X}\}$ for cLDPC codes.

**Fig. 3:**
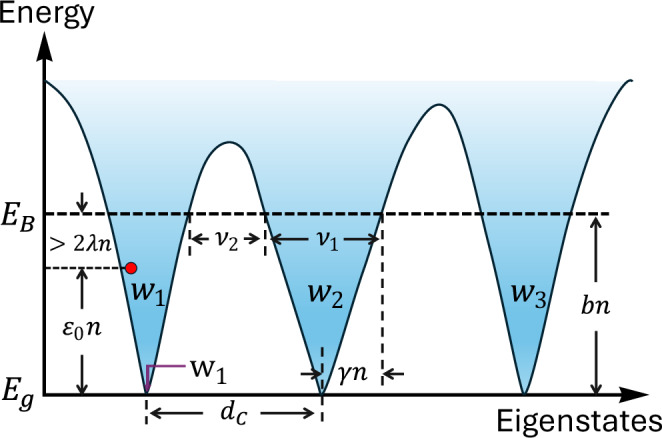
Illustration of the clustering property in classical/quantum low-density parity-check (LDPC) codes with linear soundness, where the blue area denotes all the eigenstates. Below the barrier energy *E*_*B*_ (*bn* above ground energy *E*_*g*_), eigenstates are separated into different clusters *w*_1_, *w*_2_, etc, with maximum cluster diameter *ν*_1_≤2*γn* and minimum inter-cluster distances *ν*_2_≥*d*_*C*_ − 2*γn*. According to Proposition II.1, when time-dependent perturbations with total variation bounded by *λn* are added, any initial eigenstates (e.g., the red dot in the cluster *w*_1_ near the codeword w_1_) below *E*_*B*_ − 2*λn* are localized within their clusters for an exponentially long time.

To efficiently recover the original state, a preferred property for error-correcting codes is the linear soundness

5 $${{{\bf{H}}}}({\left\vert {{{\rm{v}}}}\right\rangle }_{{{{\rm{w}}}}})\ge \alpha {{{\bf{D}}}}({\left\vert {{{\rm{v}}}}\right\rangle }_{{{{\rm{w}}}}},\left\vert {{{\rm{w}}}}\right\rangle ),\,\,{\mbox{if}}\,\,{{{\bf{D}}}}({\left\vert {{{\rm{v}}}}\right\rangle }_{{{{\rm{w}}}}},\left\vert {{{\rm{w}}}}\right\rangle )\le \gamma n,$$

where **H** counts the total number of check violations in ${\left\vert {{{\rm{v}}}}\right\rangle }_{{{{\rm{w}}}}}$. This ensures that if the erroneous state violates only a few checks, it stays near the original codeword but still far from other codewords (see Fig. 3), which is desirable for efficient decoding algorithms^30^. In addition, the above property naturally gives rise to an energy window [*E*_*g*_, *E*_*B*_] with *E*_*g*_ being the ground energy and *E*_*B*_ being the top for all clusters

6 $${E}_{B}\equiv {E}_{g}+bn,\,b\equiv 2\alpha \gamma,$$

where we can define the energy barrier as *B* ≡ *E*_*B*_ − *E*_*g*_ = *bn*. In the energy window [*E*_*g*_, *E*_*B*_], one can prove that the clustering property 1 is satisfied with

7 $${\nu }_{1}=2\gamma n,\,{\nu }_{2}={d}_{C}-2\gamma n,$$

where the code distance *d*_*C*_ > 2*γn* is a natural decodability constraint. Here, each cluster *w* is defined by eigenstates below energy *E*_*B*_ and within distance *ν*_1_ from a codeword $\left\vert {{{\rm{w}}}}\right\rangle$. This induces a linear energy barrier in the configuration space and provides stability against numerous kinds of perturbations (see Table 2). Below, we mainly discuss two common scenarios: 1. dynamical stability under unitary evolution; 2. thermodynamic stability under quantum channels, or equivalently, static Lindbladian evolution.

Dynamical localization is highly nontrivial but can exist in spin glass^48,49^. Most of the existing works discuss dynamics under static perturbations. Here, we focus on time-dependent perturbations, which is a fundamentally different scenario but has easy reductions to static perturbations.

We consider a common and realistic scenario in a self-correcting quantum memory^36^ or other quantum devices: The system is initially an eigenstate $\left\vert \psi (0)\right\rangle$ with energy *E*_0_ of *H*_*C*_, a good classical or quantum LDPC code with linear soundness. The state has an energy density ${\varepsilon }_{0}\equiv \frac{{E}_{0}-{E}_{g}}{n}$ above ground energy and is close to one codeword $\left\vert {{{{\rm{w}}}}}_{0}\right\rangle$ (see Fig. 3), which represents an erroneous state with correctable errors. Then a time-dependent perturbation *V*(*t*) is added, and the state $\left\vert \psi (t)\right\rangle$ evolves under the Hamiltonian

8 $$H(t)={H}_{C}+V(t).$$

We may ask: how long can the evolving state $\left\vert \psi (t)\right\rangle$ stay near the original codeword $\left\vert {{{{\rm{w}}}}}_{0}\right\rangle$ and remain correctable? Such stability under generic time-dependent perturbations is usually considered theoretically intractable, and we are not aware of any prior theoretical study on this question. Below, we show that by applying Theorem 1, we can rigorously prove that the erroneous state remains correctable for an exponentially long time.

#### Proposition II.1

(Exponentially long dynamical localization under time-dependent perturbations)

Let $\left\vert \psi (t)\right\rangle$ be $\left\vert \psi (0)\right\rangle$ evolving under *H*(*t*) = *H*_*C*_ + *V*(*t*), where *H*_*C*_ is defined in Eq. (3) and *V*(*t*) is a (quasi-)*q*-local perturbation. Suppose $\left\vert \psi (0)\right\rangle$ is initialized at an eigenstate of *H*_*C*_ inside a cluster *w*_0_, whose energy density *ε*_0_ above the ground state satisfies *ε*_0_ < *b* − 2*λ* and *λ* is defined in Eq. (15). Then for all Cases 1-4, $\left\vert \psi (t)\right\rangle$ almost resides within the cluster *w*_0_ up to an exponentially long time $T \sim \frac{1}{\lambda }{e}^{\Omega (n)}$, with an exponentially small leakage *e*^−*Ω*(*n*)^ for all *t*≤*T*.

We present a sketch of the proof in Methods and the details of the proof in Appendix C in the Supplementary Information. The results demonstrate that quantum and classical error-correcting codes with a linear energy barrier can protect information against generic time-dependent perturbations for an exponentially long time, as long as the total variation induced by the perturbation is bounded by *λn*. This includes two common scenarios: 1. Perturbations are global but weak, such as unstable global pulses applied to every site. 2. Perturbations are local but strong, such as cosmic rays^50–52^. This property defines a stable phase that is robust against the most general and realistic time-dependent perturbations.

The results can also be directly reduced to static perturbations by setting *V*(*t*) to be fixed. In addition, if the perturbation is weaker so that eigenstate localization^33^ can appear in a cLDPC system (whether eigenstate localization can happen in qLDPC codes remains open), the dynamical localization time can become infinite (at finite *n*). We will describe the details in Methods and provide rigorous proofs in Appendix D in the Supplementary Information. In contrast to the original work^33^ on eigenstate localization, our methods can straightforwardly generalize the *q*-local perturbations to quasi-*q*-local.

In reality, quantum devices inevitably interact with environments and become open systems, whose equilibrium state can be described by the Gibbs state ${\rho }_{H}=\frac{1}{{{{\mathcal{Z}}}}}{e}^{-\beta H}$. For systems weakly coupled to a large and stable environment, the evolution of the density matrix can be described by a static (i.e., time-independent) Lindbladian. This can also be equivalently described by repetitively applying a Markovian quantum channel ${{{\mathcal{M}}}}$, which is more common in the design of Gibbs samplers^53–55^.

Suppose that the system is initialized at one codeword. If the evolving state quickly reaches thermal equilibrium, then all codewords will be mixed, and the encoded information will be lost. Thus, slow mixing indicates the thermodynamic stability of LDPC codes.

For LDPC codes with linear soundness, the mixing time is known to be exponentially long^13^. In addition, even if strictly *q*-local perturbations are added *H* = *H*_*C*_ + *V*_0_, the slow mixing property of Gibbs samplers persists^14,56^. We now extend the results to quasi-*q*-local perturbations via Theorem 2.

#### Proposition II.2

(Robust slow mixing of Gibbs sampler with (quasi-)*q*-local perturbations)

Let *H* = *H*_*C*_ + *V*_0_, with *H*_*C*_ defined in Eq. (3) and *V*_0_ being a (quasi-)*q*-local perturbation. If *λ* (and *q*_⋆_) defined in Cases ${2}^{{\prime} }$-${4}^{{\prime} }$ is sufficiently small, any (quasi-)*q*-local Gibbs sampler ${{{\mathcal{M}}}}$ whose steady state is *e*^−*βH*^ has an exponentially long mixing time *e*^*Ω*(*n*)^.

We show the proof in Appendix E in the Supplementary Information by invoking the quantum bottleneck theorem developed in ref. ^14^. A key step in this proof is to upper-bound the low-energy leakage in high-energy eigenstates, which can be exponentially bounded by Theorem 2.

### Localization as an algorithmic barrier in hard optimization problems

Classical optimization problems are widely studied in computer science, physics, and many other areas. Among them, many are classified as NP-hard in the worst cases. Although some NP-hard problems admit efficient approximate algorithms^57–59^, some problems in certain parameter regions, however, are difficult to find approximate solutions even in average cases. These include *p*-spin glasses^46,60,61^, max *k*-SAT^61–65^ and maximum independent sets^45,66^.

**Fig. 4:**
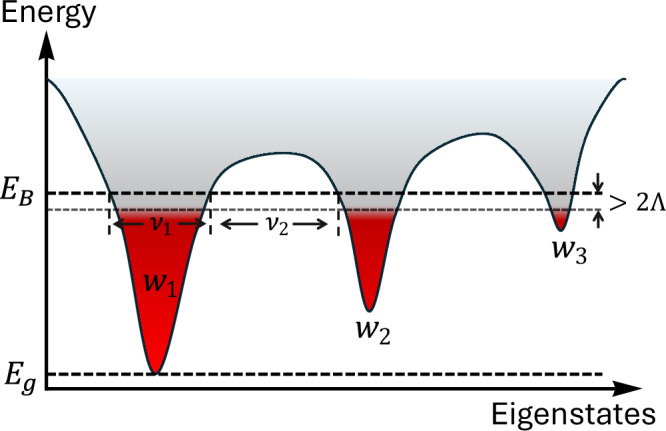
Illustration of the clustering property in hard optimization problems, where all eigenstates are simply *Z*-basis states. Below some barrier energy *E*_*B*_, eigenstates are separated into clusters, with maximum cluster diameter *ν*_1_ and minimum inter-cluster distances *ν*_2_. If the total variation of the algorithmic driving is *Λ*, then states localized below *E*_*B*_ − 2*Λ* (red zones) cannot escape from their clusters. In most cases, the number of the shallower local minima, like *w*_3_, is much larger than the deeper minima, like *w*_1_ and *w*_2_.

Such a hardness of approximation is closely related to the solution space geometry^34,67^ of these problems (see Fig. 4), which is similar to that of error-correcting codes: States with energy below a certain threshold are well-separated into clusters. A way to describe such a feature is the overlap gap property (OGP)^34^: suppose that the optimal value for an optimization problem ${{{\mathcal{L}}}}({{{\bf{z}}}})$ is ${E}_{g}={{\min }_{{{{\bf{z}}}}}}{{{\mathcal{L}}}}({{{\bf{z}}}})$, and let **z**_1_ and **z**_2_ be two near-optimal configurations such that ${{{\mathcal{L}}}}({{{{\bf{z}}}}}_{1/2})\le {E}_{g}+\tilde{B}$. The OGP requires that **D**(**z**_1_, **z**_2_) be either less than ${\tilde{\nu }}_{1}$ or more than ${\tilde{\nu }}_{2}$, where $0\le {\tilde{\nu }}_{1} < {\tilde{\nu }}_{2}$. For most models^60,61,66^, ${\tilde{\nu }}_{1}$ will approach 0 near the ground states, so that one can achieve ${\tilde{\nu }}_{2} > 2{\tilde{\nu }}_{1}$ by choosing a proper $B < \tilde{B}$. In this case, one can verify that the clustering property 1 is always satisfied with ${\nu }_{1}={\tilde{\nu }}_{1}$ and ${\nu }_{2}={\tilde{\nu }}_{2}$ in the energy window [*E*_*g*_, *E*_*B*_] with *E*_*B*_ ≡ *E*_*g*_ + *B*.

Quantum algorithms that try to solve these hard optimization problems have been extensively studied^68–77^ in both theoretical and experimental works. In this section, we focus on a class of quantum algorithms that use Hamiltonian evolution for solution search and discuss the effects of a clustered solution space on these algorithms. For a classical (unconstrained) binary optimization problem ${{\min }_{{{{\bf{z}}}}}}{{{\mathcal{L}}}}({{{\bf{z}}}})$, we can always replace the classical binary variables z with $\frac{1-Z}{2}$ and define the corresponding Pauli *Z*-basis qubit Hamiltonian ${H}_{{{{\mathcal{L}}}}}$, whose ground state encodes the optimal solution of ${{{\mathcal{L}}}}$. The algorithmic Hamiltonian we consider is defined by

9 $$H(t)={H}_{{{{\mathcal{L}}}}}+V(t),$$

where *V*(*t*) is a *q*-local Hamiltonian aiming to drive the evolving state to near-ground states of ${H}_{{{{\mathcal{L}}}}}$, and often vanishes at the end of the algorithm. Many generic quantum algorithms contain this type of evolution, such as quantum (diabatic) annealing^78,79^, quantum adiabatic algorithm^68,80,81^, and quantum Hamiltonian descent^82^.

The general performance analysis for these algorithms has not been established. Our results on dynamical localization partially fill the gap in the region where changes in *V*(*t*) are sufficiently small. In this case, any near-optimal solution is stuck in each cluster and cannot be improved by any algorithmic evolution *H*(*t*) with a total variation less than *Λ* up to a superpolynomial time. This can be summarized as:

#### Proposition II.3

(Freezing of solutions) If the total variation *Λ* (defined in Eq. (15)) of the algorithmic Hamiltonian *H*(*t*) (defined in Eq. (9)) in a time interval is below *B*/2 and ∥*H*(*t*)∥_loc_≤*M*, then any *Z*-basis state initially inside a cluster *w*_0_ with energy below *E*_*B*_ − 2*Λ* is localized inside *w*_0_ up to $T \sim \frac{1}{\Lambda }{e}^{\Omega (\Lambda /M)}$.

The results can be directly inferred from the dynamical localization II.1 and rule out the success of those quantum algorithms without sufficiently large variations to find the near-optimal results. Here, we can choose the problem of finding the (near) ground state energy of a *q*-spin glass^61^ on a random regular *p*-degree *q*-uniform hypergraph *G* via the adiabatic algorithm as an example, whose problem Hamiltonian can be written as

10 $${H}_{{{{\mathcal{L}}}}}\equiv {\sum}_{\langle {i}_{1},{i}_{2},...{i}_{q}| \in \rangle G}{J}_{{i}_{1},{i}_{2},...{i}_{q}}{Z}_{{i}_{1}}{Z}_{{i}_{2}}...{Z}_{{i}_{q}},$$

where *q*≥4 is the size of an hyperedge and each ${J}_{{i}_{1},{i}_{2},...{i}_{q}}$ can be chosen independently and randomly from { − 1, 1}. It was demonstrated in ref. ^61^ that the model has the clustering property in a linear energy window [*E*_*g*_, *E*_*g*_ + *B*] near the ground states. The corresponding adiabatic algorithm can be given via the following time-dependent Hamiltonian,

11 $$H(t)=s(t){H}_{{{{\mathcal{L}}}}}+\left(1-s(t)\right){H}_{M},$$

where *H*_*M*_ is a coupling Hamiltonian whose ground state can be easily prepared, e.g., *H*_*M*_ = ∑_*j*_*X*_*j*_, and *s*(*t*) is a monotonic function increasing from 0 to 1. As $\parallel {H}_{{{{\mathcal{L}}}}}{\parallel }_{{{{\rm{loc}}}}}=p$ (the degree of the hypergraph), the parameters *M* and *Λ*, *B* ~ *Θ*(*n*) are still well defined in Theorem 1 and Proposition II.1, where the total variation *Λ* for the evolution from some finite *s* > 0 to 1 can be defined as $\Lambda=\frac{1-s}{s}\parallel {H}_{M}{\parallel }_{{{{\rm{X}}}}}$.

In^83^, it was demonstrated from perturbative arguments that at some vanishing (when *n* → *∞*) $\tilde{\lambda }$ the *H*(*t*) will have an exponentially small gap between ground states and excited states in the worst cases, which means adiabatic algorithms can take an exponentially long time to find the exact ground state of a worst-case ${H}_{{{{\mathcal{L}}}}}$. Since finding the exact minimum is unlikely, if we reduce our goal from exact ground states to low-energy states with energy much below *E*_*B*_, can evolution with polynomial time produce good results? Unfortunately, our results give a negative answer: For *s*(*t*)≥*s*_⋆_ and ${\lambda }_{\star }\equiv \frac{1-{s}_{\star }}{{s}_{\star }}\ll 1$ is small but still *Θ*(1), which is far before the vanishing $\tilde{\lambda }$ in^83^, the system already fall into the region where any solutions below *E*_*B*_ − 2*λ*_⋆_*n* are frozen inside their clusters and cannot directly tunnel between each other (see Fig. 4). In this case, not just the exact optimum in a worst-case ${H}_{{{{\mathcal{L}}}}}$, even approximate optimal solutions in a typical-case ${H}_{{{{\mathcal{L}}}}}$ become hard to find. On the other hand, when *s*(*t*) is close to 0, the corresponding total variation of the adiabatic driving is typically not small, whose effects may not fall into the scope of our theorems.

Thus, if the state before *s*_⋆_ does not contain enough weights in near-optimal clusters, they cannot be improved by the evolution from *s*_⋆_ to 1 within polynomial time. On the other hand, if we do find some near-optimal solutions before *s*_⋆_, dynamical localization can protect good solutions from escaping. This means that even if we rapidly drive *s*(*t*) from *s*_⋆_ to 1, those good solutions remain close to their local minima and can be efficiently recovered by classical local search methods.

We leave open the possibility of whether those solutions in near-optimal (deep) clusters can have significant weights in the evolving states before *s*_⋆_, and add some intuitive remarks here. First, due to the locality of the coupling *H*_*M*_, the evolving state cannot fully explore the low-energy solution subspace of ${H}_{{{{\mathcal{L}}}}}$ without reaching high-energy solutions. Within those high-energy solutions slightly above *E*_*B*_, typically there are exponentially more of them connected to shallow clusters than those connected to deep clusters^46^. Thus, we expect that it is generally hard for both classical and quantum algorithms to significantly explore inside the deep clusters to find good solutions. On the other hand, given the flexibility in choosing *H*_*M*_ and evolution paths, it is also hard to rigorously rule out all possible polynomial-time adiabatic algorithms to find good solutions. Even if we consider a weaker task to rule out the possibilities to find the exact optimum, this remains extremely hard. If one can prove no polynomial-time adiabatic algorithms are capable of finding the exact optimum, given their equivalence to circuit-based quantum algorithms^84,85^, it is equivalent to showing that a problem can be in QMA^86^ but not in BQP^87^, which would be a groundbreaking result in quantum complexity theory.

Nevertheless, regardless of its performance before *s*_⋆_, if our goal is to find classically inaccessible solutions much below *E*_*B*_, the late-time evolution of *s*(*t*) from *s*_⋆_ to 1 is ineffective, in the sense that it will neither improve nor worsen the output states in polynomial time. For such a goal, unless we are given an exponentially long runtime, we should accelerate the late-time evolution regardless of the possible exponentially small gap within this time interval, or we can simply stop the evolution at *s*_⋆_, which will not affect much the quality of the final output states.

### Potential experimental verification

We briefly discuss how to experimentally detect the energy-space localization and its consequences. Directly probing the exponentially small leakage outside the energy window ${{{{\mathcal{E}}}}}_{0}(d)$ is usually infeasible in practice. Thus, we need the unusual stability inferred from energy-space localization in systems with energy barriers to detect and confirm it. One approach is to test the stability of a Hamiltonian built from LDPC codes or hard optimization problems under time-dependent perturbations.

For cLDPC codes specifically, the experimental procedure is simpler. We can prepare the initial state to be any codeword for simplicity, which is in the computational basis, then add a generic time-dependent perturbation with bounded total variation. After a long time, we can measure in the *Z* basis and check whether we obtain: (1) the number of violated checks is bounded; (2) the output bit string is close to the initial state in terms of average Hamming distance. Similar experiments can also be performed on models built from hard optimization problems, where one can prepare the initial state to be an approximate classical solution close to optimum.

For qLDPC codes, detecting the localization is more complicated, as the state distance here is hard to directly measure. We can initialize the system at a logical *Z* state for simplicity, and evolve the perturbed system for a long time. We repeat this with identical perturbations for multiple runs and obtain many copies of the erroneous states after long-time perturbation. Then, (1) we measure the stabilizers to see the syndromes, (2) we apply the decoder to correct them, and (3) we measure the logical *Z* operators to see if it remains the original codeword. Our energy-space localization predicts that the number of syndromes will saturate to  ≲ *λn* even after a very long time, and the corrected logical *Z* values remain the same as the initial states with high probability, which one can implement and verify with sufficient statistics.

To distinguish this new stability from other stable phases like MBL and prethermalization, the generic time-dependency of the perturbations is essential. To fully rule out the effects from prethermalization or other localization mechanisms, one can implement many time-dependent perturbations and evolve the system for at least *T* ≫ *O*(*n*) time and average over the results. We expect that the effects from energy-localization can still survive while other mechanisms cannot.

We remark that existing constructions of good LDPC codes and hard optimization problems all require long-range connectivity of qubits, which is not easy to realize via native local couplings of qubits. However, nowadays such long-range couplings can be experimentally implemented in quantum platforms of trapped ions^88–90^ and neutral atom systems^91,92^. For models built from hard optimization problems, another way to circumvent long-range interactions is to encode them in equivalent 2D problems^93^. Therefore, the dynamics of the models discussed can in principle be simulated on these experimental platforms, but the intrinsic noise inside current quantum computers limits the evolution time of those simulations.

## Discussion

In this work, we have introduced and proved the energy-space localization as a universal property of time-dependent *q*-local Hamiltonians (Theorem 1). The static energy-space localization stated in Theorem 2 can be regarded as a special limit of the dynamical case in Theorem 1. These theorems, serving as both fundamental theoretical results and versatile mathematical tools, have enabled us to demonstrate wide applications in proving the stability of systems with extensive energy barriers, such as in certain LDPC codes and hard optimization problems.

The energy-space localization also allows us to study generic quantum evolution in the lens of energy changes. In the thermodynamic limit, the exponentially decaying contributions from eigenstates with *D* > *Λ* (see Methods) naturally reduce to the classical constraint due to energy conservation, demonstrating a provable quantum-classical correspondence. The property also holds ubiquitously in qubit and fermionic systems, as long as their local norms are bounded, except for bosonic systems, where the local norms are unbounded.

Using energy-space localization, we have shown that systems with extensive energy barriers exhibit extremely stable phases, even under time-dependent perturbations. Among the various stabilities demonstrated for LDPC codes (as summarized in Table 2), the exponentially long dynamical localization against generic time-dependent perturbations stands out for its theoretical novelty and practical implications. This result not only establishes proof of stability in a previously considered intractable time-dependent setting but also provides a theoretical foundation on how error-correcting codes protect information against realistic time-varying noise. We have also extended other previously known stabilities of LDPC codes to quasi-*q*-local perturbations. On the other hand, for hard optimization problems, we proved that the evolving state under an algorithmic driving Hamiltonian becomes trapped inside local minima for an exponentially long time if the total variation of the driving is not sufficiently large. In summary, our theoretical framework rigorously connects the intrinsic property of quantum evolution with the stability of error-correcting codes and the algorithmic hardness of optimization problems from the perspective of the energy space.

We have also discussed how to experimentally probe the unusual localization. Key difficulties in these experiments are the requirements for long-range qubit connectivity and long-time unitary evolution. Although long-range connectivity can indeed be realized in many quantum platforms, long-time evolution is difficult to simulate due to noise and decoherence.

Our results also suggest several promising future research directions. Firstly, since our leakage bounds apply to generic systems, they can be further improved in specific systems, such as mean-field models (e.g., SYK models^94^) and in specific circumstances, such as short-time evolution and the low-energy subspace. Secondly, the bounds are closely related to the instantaneous higher moments of a Hamiltonian and its expectations of the nested commutators, which also appear in the study of Lieb-Robinson bounds^95^, operator growth^96,97^, and Krylov subspace methods^98^, where many well-established tools in these areas might be combined with our energy-space localization to obtain better bounds. Our theoretical framework may also help establish deeper connections among them, e.g., in quench dynamics.

## Methods

In this section, we provide some brief but intuitive descriptions of the techniques used in this work and refer the readers to the Supplementary Information for detailed and rigorous proofs. First, we discuss some simple extensions and corollaries from energy-space localization in more generalized scenarios. Second, we briefly describe how to reduce the time-dependent energy-space localization (Theorem 1) to static cases (Theorem 2). Third, we give an intuitive description of the techniques used in proving dynamical localization of LDPC codes. Fourth, we briefly show that if the perturbed eigenstates remain localized under static perturbations, which only appears in cLDPC codes (and classical hard optimization problems), the dynamical localization time can be infinitely long. Finally, we illustrate with a simple non-interacting model that can exhibit all the stabilities discussed in this work to show that they arise purely due to the energy barriers. This is made possible by restricting the dynamics in a clustered subspace via symmetry induced from a hard optimization problem.

### Extensions for energy-space localization

The key step (see Appendix B in the Supplementary Information) to prove the energy-space localization (Theorem 1) is to bound the growth of arbitrary 2*k*-th moments $\langle \psi (t)| {(H(t)-{E}_{0})}^{2k}| \psi (t)\rangle$. The bounds are closely related to the growth of the nested commutators ${{{{\rm{ad}}}}}_{H(t)}^{m}(H^{\prime} (t))$, where we denote ad_*H*_(*V*) ≡ [*H*, *V*] and ${{{{\rm{ad}}}}}_{H}^{m}(V)\equiv [H,{{{{\rm{ad}}}}}_{H}^{m-1}(V)]$ for brevity. Thus, our results apply to any *H*(*t*) whose ${{{{\rm{ad}}}}}_{H(t)}^{m}(H^{\prime} (t))$ follows similar scalings, which can be summarized in the following corollary:

#### Corollary 1

(Bounds for energy-space localization from nested commutators, informal) For any *H*(*t*) with ∫∥d*H*∥_*X*_≤*λn*, if the norm of the nested commutators $\left\Vert {{{{\rm{ad}}}}}_{H(t)}^{m}\left(\frac{H^{\prime} (t)}{\parallel H^{\prime} (t){\parallel }_{{{{\rm{X}}}}}}\right)\right\Vert$ can be uniformly bounded by a series *F*_*m*_ ~ *O*(*m*!*Δ*^*m*^) that grows at most factorially with *m*, the leakage outside the energy window ${{{{\mathcal{E}}}}}_{0}(d)$ can be bounded by ${\epsilon }_{\lambda,\Delta }^{(1)}(d)$. If *F*_*m*_ ~ *O*(*Δ*^*m*^) grows at most exponentially with *m*, the bound can be improved to ${\epsilon }_{\lambda,\Delta }^{(2)}(d)$.

The Corollary can be inferred from the proofs in Appendix B of the Supplementary Information. Intuitively, since commuting modifications of *H*(*t*) will not mix different eigenstates in the spectrum of the unmodified Hamiltonian, the contribution of the weights outside the energy window can only come from the non-commuting terms, which can be quantified by the nested commutators ${{{{\rm{ad}}}}}_{H(t)}^{m}(H^{\prime} (t))$. As the nested commutators grow at most factorially^96,97,99^ with *m* for *q*-local Hamiltonians, our results can be applied to any *q*-local *H*(*t*) with a bounded local norm.

We can then extend the energy-space localization to further cases (i.e., Case 2–4 in the main text), where the scaling of the nested commutators remains bounded above by a factorial growth (see Appendix F of the Supplementary Information).

Although our result requires the initial state to be an eigenstate of *H*(0), it can be easily extended to general initial states $\left\vert \Psi (0)\right\rangle$, which can always be expanded $\left\vert \Psi (0)\right\rangle={\sum }_{j}{c}_{j}\left\vert {\psi }_{j}(0)\right\rangle$ in the eigenbasis of *H*(0). Taking Case 1 as an example, the final state is simply $\left\vert \Psi (T)\right\rangle={\sum }_{j}{c}_{j}\left\vert {\psi }_{j}(T)\right\rangle$, where each $\left\vert {\psi }_{j}(T)\right\rangle$ is the final state evolving from $\left\vert {\psi }_{j}(0)\right\rangle$ via *H*(*t*). Suppose that the energy window defined for each $\left\vert {\psi }_{j}(0)\right\rangle$ is ${{{{\mathcal{E}}}}}_{j}(d)\equiv [{E}_{j}-dn,{E}_{j}+dn]$, then we can bound the final weight *ϵ*_*E*_ for any energy-*E* level by

12 $${\epsilon }_{E}\le {{\inf }_{d > \lambda }}\left({\sum}_{E\in {{{{\mathcal{E}}}}}_{j}(d)}| {c}_{j}|+{\sum}_{E\notin {{{{\mathcal{E}}}}}_{j}(d)}| {c}_{j}| {\epsilon }_{\lambda,{\Delta }_{q}}^{(1)}(d)\right).$$

In addition, our method does not rely on specific scalings of the total variation or local norm; the results can easily be extended to general scalings.

#### Corollary 2

(Bounds for general scalings, informal) If *Λ* and *M* of *H*(*t*) from ∫∥d*H*∥_*X*_≤*Λ* and ∥*H*(*t*)∥_loc_≤*M* both follow general scalings with *n*, then one can choose an energy window [*E*_0_ − *D*, *E*_0_ + *D*] with *D* ≳ *Λ*, such that the leakage outside the window can be bounded at the order of *e*^−*Ω*(*Λ*/*M*)^.

We present a brief proof of this Corollary in Appendix B.3 of the Supplementary Information. We remark that Theorem 1 is its special case where *Λ* = *λn*, *D* = *dn* and *M* is *O*(1).

### Static reduction for energy-space localization

The energy-space localization from Theorem 1 can be easily reduced to static perturbations. To do the reduction, we can consider $\left\vert \psi (0)\right\rangle$ being an eigenstate of *H* = *H*_0_ + *V*_0_ that follows the evolution from *t* = 0 to *T* under the time-dependent Hamiltonian

13 $$H(t)={H}_{0}+(1-\frac{t}{T}){V}_{0}.$$

Obviously, *H*(*T*) = *H*_0_. We then take the *T* → 0^+^ limit. From the Schrödinger equation, we know that a finite quench of the Hamiltonian will not instantly change the wavefunction, so the state $\left\vert \psi ({0}^{+})\right\rangle=\left\vert \psi (0)\right\rangle$ remains the eigenstate of *H*_0_ + *V*_0_, but is now exponentially localized in the energy space of *H*_0_ by Theorem 1. Then *ϵ*^(1)^ and *ϵ*^(2)^ in Theorem 1 explicitly bound the leakage outside the energy window in the eigenbasis of *H*_0_. Since here $[V(t),H^{\prime} (t)]=0$ is always satisfied by construction, as *V*(*t*) = (1 − *t*/*T*)*V*_0_, we no longer need the general Case 1 but only need Cases 2, 3, and 4. In this way, the Cases 2-4 can be translated into Cases ${2}^{{\prime} }$-${4}^{{\prime} }$, respectively.

### Sketch of the proof of dynamical localization of LDPC codes

Here we give an intuitive proof sketch for dynamical localization (Proposition II.1) of LDPC codes and leave the full rigorous one to Appendix C of the Supplementary Information. For strictly *q*-local *V*(*t*), since its matrix elements for eigenstates of *H*_*C*_ (the Hamiltonian of an LDPC code) from different clusters are exactly zero, the evolving state cannot tunnel to other clusters without reaching a higher-energy eigenstate of *H*_*C*_. Thus, if we can show that the high-energy weights *ϵ*_>_ in the evolving state $\left\vert \psi (t)\right\rangle$ in the eigenbasis of *H*_*C*_ are exponentially small at any time within [0, *T*], then the whole leakage can be upper-bounded roughly by *λnTϵ*_>_.

To bound the high-energy weights in the eigenbasis of *H*_*C*_ at time *t*_1_, we can invoke Theorem 1 for an extended evolution for $\left\vert \tilde{\psi }(t)\right\rangle$ under

14 $${H}_{{{{\rm{ext}}}}}(t)=\left\{\begin{array}{ll}{H}_{C}\hfill\quad &\,{{{\rm{for}}}}\,t={0}^{-},\hfill\\ {H}_{C}+V(t)\quad &\,{{{\rm{for}}}}\,0\le t\le {t}_{1},\\ {H}_{C}\hfill\quad &\,{{{\rm{for}}}}\,t={t}_{1}^{+},\hfill\end{array}\right.$$

where we set $\left\vert \tilde{\psi }({0}^{-})\right\rangle=\left\vert \psi (0)\right\rangle$. We also define that the *H*_ext_(*t*) at *t* = 0^−^, 0 and ${t}_{1},{t}_{1}^{+}$ are linearly connected (similar to Eq. (13)) and we let the $\left\vert \tilde{\psi }(t)\right\rangle$ evolve from 0^−^ to ${t}_{1}^{+}$. Here, we should use the total variation of *H*_ext_(*t*) instead of *H*(*t*) and define *λ* for *q*-local perturbation in Cases 1-3 as

15 $$\parallel V(0){\parallel }_{{{{\rm{X}}}}}+\int_{0}^{T}\parallel V^{\prime} (t){\parallel }_{{{{\rm{X}}}}}\le \lambda n\equiv \Lambda .$$

For quasi-*q*_⋆_-local *V*(*t*) in Case 4, we define *λ* as

16 $$\parallel V(0){\parallel }_{q{{{\rm{-X}}}}}+\int_{0}^{T}\parallel V^{\prime} (t){\parallel }_{q{{{\rm{-X}}}}}\le \lambda n\equiv \Lambda .$$

From Schrödinger’s equation, we know that $\left\vert \tilde{\psi }({t}_{1}^{+})\right\rangle$ from *H*_ext_(*t*) is the same as $\left\vert \psi ({t}_{1})\right\rangle$ from *H*(*t*). Thus, we can bound the high-energy leakage of $\left\vert \psi ({t}_{1})\right\rangle$ in the eigenbasis of *H*_*C*_ at any time *t*_1_, at the cost of adding a factor 2 to the perturbation strength.

Therefore, as long as *λ* < (*b* − *ε*_0_)/2, the leakage to high-energy states can be bounded by ${\epsilon }_{2\lambda,{\Delta }_{q}}^{(1)}(b-{\varepsilon }_{0})$ (and can be improved to ${\epsilon }_{2\lambda,{\Delta }_{q}}^{(2)}(b-{\varepsilon }_{0})$ if *V*(*t*) satisfies the condition in Case 3), which is exponentially small and is denoted by *e*^−*ξn*^. Thus, we can choose $T\lesssim \frac{1}{\lambda n}{e}^{{\xi }_{1}n}$ with any *ξ*_1_ < *ξ*, and bound the whole leakage by ${e}^{-(\xi -{\xi }_{1})n}$.

### Eigenstate localization and infinite-time dynamical localization

In the main text, we show that classical and quantum LDPC codes with linear energy barriers can remain localized for an exponentially long time against time-dependent perturbations, which can also be applied to static perturbations. Here, we show that the localization can be infinitely long if the static perturbation strength is weak enough, due to eigenstate localization^33^.

This property was recently discovered^33^ in certain cLDPC codes. By invoking Theorem 2, we reproduce the results and generalize them to include quasi-*q*-local perturbations. Specifically, we consider the following Hamiltonian

17 $$H={H}_{C}+{V}_{0}+{H}_{{{{\rm{d}}}}},$$

where *H*_*C*_ is a cLDPC Hamiltonian with linear soundness defined above, *V*_0_ is a (quasi-)*q*-local perturbation with ∥*V*∥_(*q*−)*X*_≤*λn*, and *H*_d_ is a vanishingly small detuning term and we can choose ${H}_{{{{\rm{d}}}}}\equiv \frac{1}{{n}^{2}}{\sum }_{j}{h}_{j}^{z}{Z}_{j}$ with *O*(1) randomly chosen ${h}_{j}^{z}$. We claim that

#### Proposition IV.1

(Eigenstate localization with (quasi-)*q*-local perturbations, informal)

For the *H* defined above ((17)), if *λ* (also *q*_⋆_ for quasi-*q*_⋆_-local) is sufficiently small, each perturbed eigenstate $\left\vert \psi \right\rangle$ of *H* with energy below *E*_*B*_ is exponentially localized near one codeword $\left\vert {{{\rm{w}}}}\right\rangle$, with leakage roughly bounded by *ϵ*_>_/*δ*_*W*_ defined below.

We show explicit bounds, with the proof detailed in Appendix D of the Supplementary Information. Here *ϵ*_>_ is roughly the leakage to higher-energy ( > *E*_*B*_) eigenstates up to an energy scale, which can be exponentially bounded by Theorem 2. The *δ*_*W*_ is roughly the minimum detuning from *H*_d_ between different clusters. Thus, the key step of the proof is to ensure that the detuning *δ*_*W*_ is much larger than the leakage *ϵ*_>_. It turns out that for cLDPC codes, *δ*_*W*_ can be lower bounded by *e*^−2*n*^ with high probability, while for qLDPC codes, it is unlikely to lower bound *δ*_*W*_. This is because the ground state degeneracy of good qLDPC codes is stable^40,41^ against any local perturbations.

The eigenstate localization can also affect the dynamical localization time. Although the exponentially long localization time in Proposition II.1 still works here, it can be improved to infinitely long for the static Hamiltonian (17) due to eigenstate localization.

#### Proposition IV.2

(Infinitely long dynamical localization with static (quasi-)*q*-local perturbation)

Let $\left\vert \psi (t)\right\rangle$ be initialized in one cluster *w*_0_ and evolve under the static Hamiltonian: *H* = *H*_*C*_ + *V*_0_ + *H*_*d*_. If *λ* (and *q*_⋆_ in quasi-*q*_⋆_-local perturbations) is sufficiently small, the state resides almost within *w*_0_ for infinitely long time, with a leakage bounded by *e*^−*Ω*(*n*)^.

We provide the proof in Appendix C.2 of the Supplementary Information. Intuitively, if the perturbation is sufficiently weak, we can prove that the majority of $\left\vert \psi (0)\right\rangle$ comes from the localized eigenstates inside *w*_0_. Since weights on eigenstates are conserved at any time of the evolution under the time-independent Hamiltonian, the leakage outside *w*_0_ can be bounded for all time.

### Symmetry-protected localization from constraint optimization problems

To demonstrate that all the stabilities discussed in LDPC codes solely arise from the clustered energy landscape, we give an example that a trivial non-interacting Hamiltonian can have those stabilities if the perturbations respect some symmetries so that the evolution is restricted to some subspaces defined from hard constraint optimization problems.

Specifically, we can consider the maximum independent set (MIS) of a random *p*-regular graph *G*: finding the largest subset of vertices that does not contain any edges between them. The quantum Hamiltonian for the cost function ${{{\mathcal{L}}}}$ is a simple non-interacting Hamiltonian counting the (minus of) vertex number

18 $${H}_{{{{\rm{V}}}}}\equiv -{\sum}_{j}\frac{1-{Z}_{j}}{2}.$$

Here, we use the *z* = − 1 as an occupied vertex. We can also define *H*_E_(*G*) for the graph *G* to count the edge number of *Z*-basis states

19 $${H}_{{{{\rm{E}}}}}(G)\equiv \frac{1}{4}{\sum}_{\langle ij| \in \rangle G}(1-{Z}_{i})(1-{Z}_{j}).$$

Then the MIS corresponds to the ground state of *H*_V_ in the zero-subspace of *H*_E_(*G*). The model also has the clustering property^45,66^ in a linear energy window [*E*_*g*_, *E*_*g*_ + *B*].

Therefore, the non-interacting *H*_V_ should enjoy all the stability properties discussed in Sec. II, if the perturbation *V* leaves the ground state subspace of *H*_E_ invariant (this requirement is weaker than [*V*, *H*_E_] = 0). One choice of such a perturbation *V* can be

20 $$V(t)=\lambda (t){\sum}_{i}{\prod}_{j,\langle ij| \in \rangle G}{P}_{j}^{z}{X}_{i},$$

where ${P}_{j}^{z}=\frac{1+{Z}_{j}}{2}$ is the projector to state $\left\vert 0\right\rangle$ for qubit *j*. Similar interactions also appear in the PXP model^100^, which exhibits many-body quantum scars^100,101^ when *G* is some bipartite graph different from our case. In such a type of *V*, if the initial state is in the ground subspace of *H*_E_ and has sufficiently low energy in *H*_V_, it is robust against all kinds of perturbations as discussed in the case of LDPC. One can also choose ${H}_{{{{\mathcal{L}}}}}={H}_{{{{\rm{E}}}}}(G)$ and set *V* to respect the symmetry that conserves the total vertex number (i.e., [*V*, *H*_V_] = 0). Such a symmetry-preserving particle number is more common, and one can verify that its energy landscape exhibits a clustering property in a linear energy window if choosing a vertex number near MIS size; thus, it is also stable against all *q*-local symmetry-respecting *V*.

## Supplementary information

Supplementary Information

Description of Additional Supplementary Files

Supplementary Data 1

Transparent Peer Review file

## Data Availability

The data for Fig. 1 can be generated by the Mathematica codes in Supplemental Data 1.

## Code availability

The code used to generate Fig. 1 are provided in Supplementary Data 1.
