# Supplementary material for: Universal energy-space localization and stable quantum phases against time-dependent perturbations
Source: Nat Commun. 2026 Jul 14;17:8643. doi: 10.1038/s41467-026-75465-z (PMC13486644; doi:10.1038/s41467-026-75465-z)
Supplement: Supplementary file 1 [file 41467_2026_75465_MOESM1_ESM.pdf]

Supplementary Information

APPENDIX CONTENTS

A. Definition of different norms and total variations S2

B. Proof of the energy-space localization S3
1. Main proof for Cases 1, 2, 4 S4
2. Improved bounds for Case 3 S7
3. Bounds for perturbations of general scalings S8
4. Properties of $f_k^{(1)}$ S9
5. Properties of $f_k^{(2)}$ S10

C. Proof of the dynamical localization S11
1. Exponentially long time dynamical localization S13
2. Infinitely long time dynamical localization in cLDPC codes S14

D. Proof of the eigenstate localization S17
1. Eigenstate localization in cLDPC codes with strictly q-local perturbations S18
2. Eigenstate localization in cLDPC codes with quasi-q-local perturbations S19
3. Discussions on Eigenstate localization in qLDPC codes S20
4. Proof of Lemma D.1 S21
5. Proof of Lemma D.2 S22

E. Proof of the slow mixing of Gibbs samplers S23
1. Strictly q-local perturbations S24
2. Quasi-q-local perturbations S25

F. Some bounds for nested commutators S25
1. H is p-local and V is q-local S26
2. H is mutually commuting and V is q-local S27
3. H is mutually commuting and V is quasi-q-local S28

Overview of the Supplementary Information

In this supplemental material, we will show formal statements of theorems, propositions, and notations mentioned or used in the main text, as well as their detailed proofs. Appendix A gives all the definitions and properties of the norms and total variations used in this work.

In Appendix B, we give a formal statement of energy-space localization (Theorem 1), and show the detailed proofs for all cases defined in the main text. The main technique is to construct a recurrence relation for the arbitrary k-th central moment of energy, so that their bounds can be obtained recursively. In bounding the growth of the k-th central moment, we relate it to the growth of nested commutators of a q-local Hamiltonian, whose bounds are obtained with more detail in Appendix F.

In Appendix C, we prove that for systems with linear energy barriers, initial states inside one cluster are localized for an exponentially long time under generic time-dependent perturbations. Such an exponentially long-time dynamical localization only requires the linear energy barrier in the clustered solution space, regardless of whether the perturbed eigenstates are localized or not. If the perturbed eigenstates are indeed localized, we also discuss the possibility of infinite-time dynamical localization, which can only appear under static perturbations. This infinite-time dynamical localization only works when the system exhibits the eigenstate localization under static perturbations, which was originally introduced in [1] for static, strictly q-lcoal perturbations, and will be discussed and extended to quasi-q-local in Appendix D.

In Appendix D, we prove that for systems with linear energy barriers under static (quasi)-q-local perturbations, the perturbed eigenstates are localized inside the original clusters. Such eigenstate localization under static strictly q-local perturbation has already been obtained in [1]. A key technique in [1] is rewriting the perturbed Hamiltonian in a tri-diagonal form to bound the high-energy contribution in perturbed eigenstates. However, this method is hard to extend to quasi-q-local cases, as quasi-q-local terms cannot be written in tri-diagonal forms in the unperturbed basis. To resolve this, we replace the method with our Theorem 2 to bound the high-energy leakage, which can easily extend

to quasi-q-local cases, and adapt the rest of procedures in [1] to generalize the eigenstate localization to quasi-q-local perturbations.

In Appendix E, we prove that for systems with linear energy barriers under static (quasi)-q-local perturbations, their corresponding Gibbs samplers require an exponentially long time to reach equilibrium. Such slow-mixing was also obtained in [2, 3] when the perturbation is strictly q-local. The similar tri-diagonal technique to [1] is used in [2] to bound the high-energy and low-energy contributions in perturbed eigenstates. As previously mentioned, this method is hard to generalize to quasi-q-local cases. To resolve this, we replace their method with our Theorem 2 and adapt the quantum bottleneck theorem developed in [2] to generalize the results to quasi-q-local perturbations.

In Appendix F, we provide some simple proofs to bound the growth of nested commutators in different cases, whose growth is closely related to the tail bound in energy-space localization proved in Appendix B. The growth of nested commutators is well-known in studies on operator growth [4, 5], Floquet-Magnus theory [6], and ETH [7]. We briefly derive them in Appendix F for the sake of technical completeness and to maintain consistency with our definitions and notations.

Appendix A: Definition of different norms and total variations

In this work, we define various norms and total variations for mathematical convenience. Here, we summarize them with their definitions and properties.

We denote $\|H\|$ as the operator norm of a Hamiltonian H, i.e.,

$$\|H\| \equiv \sup_{|\psi\rangle \neq 0} \frac{\|H\,|\psi\rangle\,\|}{\|\,|\psi\rangle\,\|}. \tag{A1}$$

Upon this, we define the termwise total norm $\|\cdot\|_{\mathrm{X}}$ as

$$\|H\|_{\mathrm{X}} \equiv \sum_A \|h_A\|, \quad \text{for } H = \sum_A h_A, \tag{A2}$$

where we decompose H into local terms and each h_A is supported on a region A. Note that one may have different ways of decomposing the total H, e.g., coarse-graining, so the termwise norm is not generally unique. Any reasonable decomposition should suffice. Moreover, for qubit Hamiltonians, we can always choose decomposition into Pauli operators to ensure uniqueness. With the decomposition fixed, we can define the termwise total variation $\mathbf{V}_{\mathrm{X}}$ for a time-dependent $H(t)$ from $t = 0$ to T as

$$\mathbf{V}_{\mathrm{X}}(H(t); [0, T]) \equiv \int_0^T \|H'(t)\|_{\mathrm{X}} \mathrm{d}t \equiv \int_0^T \|\mathrm{d}H\|_{\mathrm{X}}, \tag{A3}$$

where $T > 0$. We can rewrite $H(t)$ as $H(t) = H(0) + V(t)$. An inequality we will frequently use in this work is

$$\|V(t)\| \leq \|V(t)\|_{\mathrm{X}} = \left\|\int_0^t \mathrm{d}H\right\|_{\mathrm{X}} \leq \int_0^t \|\mathrm{d}H\|_{\mathrm{X}}. \tag{A4}$$

These are norms for global properties of a Hamiltonian. We also need a local norm to depict the local strength of a Hamiltonian. For a Hamiltonian H with decomposition $H = \sum_A h_A$ on sites i's, we define the local norm $\|\cdot\|_{\mathrm{loc}}$ as

$$\|H\|_{\mathrm{loc}} \equiv \sup_i \sum_{A, A \ni i} \|h_A\|, \tag{A5}$$

where the supremum is over all sites i. It is obvious that for a fixed decomposition $H = \sum_A h_A$, we have

$$\|H\| \leq \|H\|_{\mathrm{X}} \leq n\|H\|_{\mathrm{loc}}, \tag{A6}$$

where n is the total number of sites.

The above definition is sufficient for strictly q-local Hamiltonians. However, for quasi-q-local Hamiltonians, we need an additional parameter q to describe its decaying speed with the number of sites involved in the interactions. We define a Hamiltonian H to be quasi-q-local if it has a decomposition $H = \sum_A h_A$, where the norm of each k-local term roughly decays exponentially $e^{-k/q}$ with the locality. To give a precise definition, we define the termwise q-norm as

$$\|H\|_{q\text{-X}} \equiv \sum_A (q+1) e^{|A|/q} \|h_A\|, \tag{A7}$$

where $q > 0$ and $|A|$ counts the number of sites of the support A. Similar definitions also appear in the study on prethermalization [8–10]. We add an additional $q+1$ factor for mathematical convenience. We define H as quasi-$q_\star$-local if one can find a $q_\star$ (usually the minimum of all possible q) such that $\frac{1}{n}\|H\|_{q_\star\text{-X}}$ can be bounded by an $O(1)$ constant. It is easy to verify that given a fixed decomposition, we have

$$\|H\| \leq \|H\|_{\text{X}} \leq \|H\|_{q\text{-X}}. \tag{A8}$$

In addition, given the decomposition $H = \sum_A h_A$, if $\|H\|_{q\text{-X}} \leq \lambda n$, where λ is $O(1)$, we have

$$\sum_{A,|A|=k} \|h_A\| \leq \frac{\lambda n}{q+1} e^{-k/q}. \tag{A9}$$

This indicates that the termwise norm of k-local components of a quasi-q-local H decays exponentially with k. Similarly, we define the total variation $\mathbf{V}_{q\text{-X}}$ of quasi-q-local $H(t)$ as

$$\mathbf{V}_{q\text{-X}}\left(H(t);[0,T]\right) \equiv \int_0^T \left\|\frac{\partial H(t)}{\partial t}\right\|_{q\text{-X}} \mathrm{d}t \equiv \int_0^T \|\mathrm{d}H\|_{q\text{-X}} \tag{A10}$$

Similarly, if we define $V(t) = H(t) - H(0)$, we have the following inequality

$$\|V(t)\| \leq \|V(t)\|_{\text{X}} \leq \|V(t)\|_{q\text{-X}} \leq \int_0^t \|\mathrm{d}H\|_{q\text{-X}}. \tag{A11}$$

Appendix B: Proof of the energy-space localization

In this section, we are going to rigorously prove the Theorem 1 for Cases 1-4. The procedures for all four cases are similar, and a key step is to bound the nested commutator $\mathrm{ad}^m_{H(t)}(H'(t))$. We use a factorially growing series to bound the commutators For Cases 1, 2 and 4, and an exponential bound for Cases 3. The proofs for same-scaling commutators are similar, so we put the proofs for the two different scalings in the Appendix B 1 and B 2, respectively.

For clarification, we rewrite Theorem 1 (for Case 1 as an example) with more details. Other cases follow a similar formulation.

Theorem 1 (Energy-space localization, for Case 1 as an example)**.** For a time-dependent q-local (no geometrical constraints here) system $H(t)$ defined on a n-site lattice, we assume that its total variation of termwise norm in $t \in [0,T]$ is bounded by

$$\int_0^T \left\|\frac{\partial H(t)}{\partial t}\right\|_{\text{X}} \mathrm{d}t \leq \lambda n. \tag{B1}$$

We assume that $H(t)$ has a finite *local* norm $\|H(t)\|_{\text{loc}} \leq M$ for any t, i.e., for a partition $H(t) = \sum_A h_A(t)$, and for any t and site i

$$\sum_{A,A\ni i} \|h_A(t)\| \leq M. \tag{B2}$$

This means that all terms that have (partial) support at site i have a finite combined norm. Let a state $|\psi(t)\rangle$, initialized in any of the eigenstates of $H(0)$ with energy E_0

$$H(0)\,|\psi(0)\rangle = E_0\,|\psi(0)\rangle\,, \tag{B3}$$

evolve according to $H(t)$, i.e.,

$$i\frac{\partial}{\partial t}\,|\psi(t)\rangle = H(t)\,|\psi(t)\rangle\,. \tag{B4}$$

Then the evolving state $|\psi(t)\rangle$ at any t can be expanded in the eigenbasis of $H(t)$:

$$|\psi(t)\rangle = \sum_j a_j(t)\,|\phi_j(t)\rangle\,, \tag{B5}$$

where $\phi_j(t)$'s are the instantaneous eigenstates of $H(t)$ with energy $E_j(t)$.

We claim that $|\psi(T)\rangle$ is exponentially localized in the energy window $\mathcal{E}_0(d) \equiv [E_0 - dn, E_0 + dn]$ in the eigenbasis of $H(T)$, with leakage $\epsilon_T(d)$ bounded by

$$\epsilon_T(d) \equiv \sqrt{\sum_{j, E_j(T) \notin \mathcal{E}_0(d)} |a_j(T)|^2} \leq e^{-Cn}, \tag{B6}$$

where $d > \lambda$ is a parameter that sets the leakage width, and C is defined by

$$C \equiv \frac{\lambda}{\Delta_q} \left(\frac{d}{\lambda} - 1 - \ln \frac{d}{\lambda} \right) + o(1) \tag{B7}$$

with $d/\lambda > 1$, $\Delta_q \equiv 2qM$ and $o(1)$ being a small positive number that will vanish if $n \to \infty$. Using the definition in Eq. (1), the bound for $\epsilon_T(d)$ can be written as

$$\epsilon_T(d) \leq \epsilon^{(1)}_{\lambda,\Delta_q}(d). \tag{B8}$$

The formulations for other cases can easily be obtained by replacing the constraints on $H(t)$ accordingly, where the leakage can be bounded by $\epsilon_T(d) \leq \epsilon^{(1)}_{\lambda,\Delta_q}(d)$ for Cases 1, 2 and 4, and $\epsilon_T(d) \leq \epsilon^{(2)}_{\lambda,\Delta_q}(d)$ for Case 3. The functions $\epsilon^{(1)}_{\lambda,\Delta_q}(d)$ and $\epsilon^{(1)}_{\lambda,\Delta_q}(d)$ are explicitly stated in the main text (in Theorem 1). We show the proofs and derive these two functions below.

1. Main proof for Cases 1, 2, 4

For Cases. 1 and 2, we define the total variation *per site* at time t with $0 \leq t \leq T$ as

$$\lambda(t) \equiv \frac{1}{n} \int_0^t \left\| \frac{\partial H(\tau)}{\partial \tau} \right\|_{\mathrm{X}} \mathrm{d}\tau. \tag{B9}$$

Then it is obvious that $\lambda(t) \geq 0$ and $\lambda'(t) \geq 0$. For brevity, we define $\lambda_t \equiv \lambda(t) \leq \lambda(T) \equiv \lambda_T = \lambda$. Additionally, according to the definition, we have the following.

$$\lambda'(t) n = \left\| \frac{\partial H(t)}{\partial t} \right\|_{\mathrm{X}}. \tag{B10}$$

Similarly, for Case 4, we can define

$$\lambda(t) \equiv \frac{1}{n} \int_0^t \left\| \frac{\partial H(\tau)}{\partial \tau} \right\|_{q\text{-X}} \mathrm{d}\tau \tag{B11}$$

and obtain

$$\lambda'(t) n = \left\| \frac{\partial H(t)}{\partial t} \right\|_{q\text{-X}}. \tag{B12}$$

Starting with $H(0)\,|\psi(0)\rangle = E_0\,|\psi(0)\rangle$, we consider the growth of the expectation of $\big(H(t) - E_0\big)^{2k}$ on $|\psi(t)\rangle$. We define the shifted Hamiltonian $\tilde{H}(t) \equiv H(t) - E_0$ and the shifted eigen-energies $\tilde{E}_j(t) \equiv E_j(t) - E_0$ for convenience. Denote the following

$$g_k(t) \equiv \left\| \tilde{H}(t)^k\,|\psi(t)\rangle \right\|. \tag{B13}$$

It is obvious that $g_k(0) = 0$ and $g_k(t)^2$ gives exactly the expectation $\langle \tilde{H}(t)^{2k} \rangle$, i.e.,

$$G_{2k}(t) \equiv \langle \psi(t) | \tilde{H}(t)^{2k} | \psi(t) \rangle = g_k(t)^2. \tag{B14}$$

With instantaneous eigenstates $|\phi_j(t)\rangle$ of $\tilde{H}(t)$ with energy $\tilde{E}_j(t)$, we can expand $|\psi(t)\rangle$ as follows:

$$|\psi(t)\rangle = \sum_j a_j(t)\,|\phi_j(t)\rangle. \tag{B15}$$

We can also interpret $|\psi(t)\rangle$ as giving rise to a classical probability distribution of energies

$$\mathbf{P}(\hat{E}_t = E) = \sum_{j,\tilde{E}_j(t)=E} |a_j(t)|^2, \tag{B16}$$

where we use the $\hat{}$ to emphasize that $\hat{E}_t$ is a classical random variable. By definition, the expectation $G_{2k}(t)$ in the instantaneous eigenbasis is the same as the $2k$-moment of $\hat{E}_t$,

$$G_{2k}(t) = \mathbf{E}(\hat{E}_t^{2k}) = \sum_j |a_j(t)|^2 \tilde{E}_j(t)^{2k}, \tag{B17}$$

Thus, the far-energy contribution from eigenstates with $|\tilde{E}_j(t)| > dn$ can be upper bounded by Markov's inequality:

$$\sum_{j,|\tilde{E}_j(t)|\geq dn} |a_j(t)|^2 = \mathbf{P}(|\hat{E}_t| \geq dn) \leq \min_k \frac{G_{2k}(t)}{(dn)^{2k}}. \tag{B18}$$

Thus, by definitions we have

$$\epsilon_t(d) \leq \min_k \frac{g_k(t)}{(dn)^k}. \tag{B19}$$

Next, we are going to bound $g_k(t)$ for arbitrary k. Denoting $\langle\cdot\rangle_t \equiv \langle\psi(t)|\cdot|\psi(t)\rangle$, using the obvious fact that $\tilde{H}(t)^k$ commutes with $H(t)$, we have

$$\frac{d}{dt}\left\langle \tilde{H}(t)^{2k}\right\rangle_t = \left\langle \frac{\partial}{\partial t}\left(\tilde{H}(t)^{2k}\right)\right\rangle_t. \tag{B20}$$

For $k = 1$, we have

$$\begin{aligned}\frac{\partial}{\partial t}G_2(t) &= \langle\psi(t)|\left(\tilde{H}(t)\frac{\partial H(t)}{\partial t} + \frac{\partial H(t)}{\partial t}\tilde{H}(t)\right)|\psi(t)\rangle \\ &\leq 2g_1(t)\left\|\frac{\partial H(t)}{\partial t}\right\| \leq 2g_1(t)\lambda'(t)n.\end{aligned} \tag{B21}$$

Using $\frac{\partial}{\partial t}G_2(t) = 2g_1(t)g_1'(t)$, we have

$$g_1'(t) \leq \lambda'(t)n. \tag{B22}$$

Since $g_1(0) = 0$ and $\lambda(0) = 0$, we arrive at

$$0 \leq g_1(t) \leq \lambda(t)n. \tag{B23}$$

In the above analysis, we implicitly assume $g_1(t) > 0$ for $t > 0$ so that we can safely eliminate one $g_1(t)$ on both sides. This can be justified as follows: By definition we have $g_k(t) \geq 0$ and $g_k(0) = 0$. If there is one additional zero point $g_1(t_1) = 0$ at some t_1, it indicates that the evolving state $|\psi(t_1)\rangle$ is exactly the instantaneous eigenstate of $H(t_1)$ with energy E_0. Thus, we can bound $g_1(t)$ by integrating $\lambda'(t)n$ from 0 to t for $0 < t < t_1$ and from t_1 to t for $t > t_1$, and both of them are bounded by the integral from 0 to t (i.e., $\lambda(t)n$). Thus, the above bound holds regardless of any additional zero points of $g_1(t)$ in $[0, T]$. Therefore, we can neglect the potential zero points and eliminate the $g_1(t)$ on both sides without affecting the final bounds. This argument holds for any $g_k(t)$: As $g_k(t_1) = 0$ indicates that $|\psi(t_1)\rangle$ is an instantaneous eigenstate of $H(t_1)$ for any k, we can set t_1 as our new initial point and the bound obtained can only be smaller than that from 0 to T.

Next, we consider $k = 2$. Similarly, we have

$$\begin{aligned}2g_2(t)g_2'(t) &= \left\langle \tilde{H}(t)^3\frac{\partial H(t)}{\partial t} + \tilde{H}(t)^2\frac{\partial H(t)}{\partial t}\tilde{H}(t) + \tilde{H}(t)\frac{\partial H(t)}{\partial t}\tilde{H}(t)^2 + \frac{\partial H(t)}{\partial t}\tilde{H}(t)^3\right\rangle_t \\ &= \left\langle 2\tilde{H}(t)^2\frac{\partial H(t)}{\partial t}\tilde{H}(t) + 2\tilde{H}(t)\frac{\partial H(t)}{\partial t}\tilde{H}(t)^2 + \tilde{H}(t)^2\left[\tilde{H}(t), \frac{\partial H(t)}{\partial t}\right] + \left[\frac{\partial H(t)}{\partial t}, \tilde{H}(t)\right]\tilde{H}(t)^2\right\rangle_t \\ &\leq 4\left\|\frac{\partial H(t)}{\partial t}\right\| g_1(t)g_2(t) + 2g_2(t)\left\|\left[\tilde{H}(t), \frac{\partial H(t)}{\partial t}\right]\right\|.\end{aligned} \tag{B24}$$

Here, we need to bound the commutator $\mathrm{ad}_{H(t)}(H'(t))$. We recall $\mathrm{ad}_A(B) \equiv [A, B]$. In fact, we can show that (see Appendix F) for Cases 1 and 2:

$$\left\|\mathrm{ad}^m_{\tilde{H}(t)}\left(\frac{\partial H(t)}{\partial t}\right)\right\| \leq m!\Delta_q^m \left\|\frac{\partial H(t)}{\partial t}\right\|_{\mathrm{X}}, \tag{B25}$$

and for Case 4:

$$\left\|\mathrm{ad}^m_{\tilde{H}(t)}\left(\frac{\partial H(t)}{\partial t}\right)\right\| \leq m!\Delta_q^m \left\|\frac{\partial H(t)}{\partial t}\right\|_{q\text{-}\mathrm{X}}. \tag{B26}$$

In all cases, the RHS is $m!\Delta_q^m \lambda'(t)n$ by definition. Taking $m = 1$ and applying the bound to Eq. (B24), we have

$$g_2'(t) \leq 2\lambda(t)\lambda'(t)n^2 + \Delta_q\lambda'(t)n. \tag{B27}$$

Thus, by integration and $g_2(0) = 0$, we obtain the upper bound

$$g_2(t) \leq \lambda(t)^2n^2 + \Delta_q\lambda(t)n. \tag{B28}$$

The procedure can continue with induction. At the k-th step, we have $\frac{\partial}{\partial t}G_{2k}(t) = \left\langle \tilde{H}(t)^k \frac{\partial \tilde{H}(t)^k}{\partial t} + \frac{\partial \tilde{H}(t)^k}{\partial t}\tilde{H}(t)^k\right\rangle_t$ and

$$\frac{\partial}{\partial t}\tilde{H}(t)^k = \sum_{m=0}^{k-1} \tilde{H}(t)^{k-m-1}\frac{\partial H(t)}{\partial t}\tilde{H}(t)^m. \tag{B29}$$

Using $VH = (H - \mathrm{ad}_H) \circ V$, we get $VH^m = (H - \mathrm{ad}_H)^m \circ V$, so that

$$\begin{aligned}
\frac{\partial}{\partial t}\tilde{H}(t)^k &= \sum_{m=0}^{k-1} \tilde{H}(t)^{k-m-1}(\tilde{H}(t) - \mathrm{ad}_{\tilde{H}(t)})^m \circ \frac{\partial H(t)}{\partial t} \\
&= \frac{\tilde{H}(t)^k - (\tilde{H}(t) - \mathrm{ad}_{\tilde{H}(t)})^k}{\mathrm{ad}_{\tilde{H}(t)}} \circ \frac{\partial H(t)}{\partial t} \\
&= \sum_{m=1}^{k} (-1)^{m-1}\binom{k}{m}\tilde{H}(t)^{k-m}\mathrm{ad}^{m-1}_{\tilde{H}(t)}\left(\frac{\partial H(t)}{\partial t}\right),
\end{aligned} \tag{B30}$$

where we have used the fact that $\tilde{H}(t)$ commutes with $\mathrm{ad}_{\tilde{H}(t)}$ when treated as linear operators, so that we can manipulate them in the above equations just like classical variables. Therefore, we have obtained

$$\begin{aligned}
2g_k(t)g_k'(t) = \frac{\partial}{\partial t}G_{2k}(t) &= \left\langle \tilde{H}(t)^k \frac{\partial \tilde{H}(t)^k}{\partial t} + \frac{\partial \tilde{H}(t)^k}{\partial t}\tilde{H}(t)^k\right\rangle_t \\
&\leq 2g_k(t)\sum_{m=1}^{k}\binom{k}{m}g_{k-m}(t)(m-1)!\Delta_q^{m-1}\lambda'(t)n,
\end{aligned} \tag{B31}$$

which gives

$$g_k'(t) \leq \sum_{m=1}^{k}\binom{k}{m}g_{k-m}(t)(m-1)!\Delta_q^{m-1}\lambda'(t)n. \tag{B32}$$

Dividing both sides by Δ_q^k, we have

$$\frac{g_k'(t)}{\Delta_q^k} \leq \sum_{m=1}^{k}\binom{k}{m}\left(\frac{g_{k-m}(t)}{\Delta_q^{k-m}}\right)(m-1)!\frac{\lambda'(t)n}{\Delta_q}. \tag{B33}$$

For convenience of dealing with the right-hand side, we define a degree-k polynomial $f_k^{(1)}(x)$ for comparison with g_k, with $f_0^{(1)}(x) \equiv 1$, $f_1^{(1)}(x) \equiv x$ and the recurrence relation

$$f_k^{(1)}(x) \equiv \int_0^x \mathrm{d}s \sum_{m=1}^{k}\binom{k}{m}(m-1)!f_{k-m}^{(1)}(s). \tag{B34}$$

One can prove by induction to show that

$$g_k(t) \le \Delta_q^k f_k^{(1)}\left(\frac{\lambda_t n}{\Delta_q}\right) \tag{B35}$$

One can further verify that (see Appendix B 4)

$$f_k^{(1)}(x) = \prod_{j=0}^{k-1}(x+j). \tag{B36}$$

By setting $x = \frac{\lambda_t n}{\Delta_q}$, we have

$$g_k(t) \le \prod_{j=0}^{k-1}(\lambda_t n + j\Delta_q). \tag{B37}$$

Using the Markov inequality at $k = k_d \equiv \lceil \frac{d-\lambda_t}{\Delta_q} n \rceil$ (i.e., setting $k = k_d$ in Eq. (B19)), we have

$$\epsilon_t(d) \le \left(\frac{\Delta_q}{dn}\right)^{k_d} \frac{\Gamma\left(\frac{\lambda_t}{\Delta_q} n + k_d\right)}{\Gamma\left(\frac{\lambda_t}{\Delta_q} n\right)}, \tag{B38}$$

where $\Gamma(x)$ is the gamma function with $\Gamma(x+1) = x\Gamma(x)$. At the large n limit, we can use Stirling's formula and obtain the bound

$$\epsilon_t(d) \le \exp\left(-n\frac{\lambda_t}{\Delta_q}\left(\frac{d}{\lambda_t} - 1 - \ln\frac{d}{\lambda_t}\right) - o(n)\right), \tag{B39}$$

where the leading order of the $-o(n)$ correction is $\frac{1}{2}\ln\frac{\lambda_t}{d} < 0$. The above bound holds for any t and decays exponentially for any $d > \lambda_t$.

2. Improved bounds for Case 3

In Case 3, we assume that the $H(t)$ admits a partition into a mutually commuting core $H_C(t)$ and the remainder $V(t)$

$$H(t) = H_C(t) + V(t), \tag{B40}$$

such that $[V(t), H'(t)] = 0$ for all t and all terms in the $H_C(t) = \sum_X h_X(t)$ are mutually commuting with each other. In this case, we can use the same definition for $\lambda(t)$ and $\lambda'(t)$ as (B9) and (B10). We do not require that the k-locality of $H_C(t)$ is finite, but only require that $H'(t)$ is q-local with finite q and $\|H_C(t)\|_{\mathrm{loc}} \le M$. An example of this case is $H_C(t) = \sum_j \sigma_j^z\sigma_{j+1}^z$ (simply time independent) and $V(t) = \frac{\lambda t}{T}\sum_j \sigma_j^x$.

Following similar procedures to those in Appendix B 1, the key different step is to bound the scaling of the nested commutators $\mathrm{ad}^m_{\tilde{H}(t)}(H'(t))$. One can verify that the nested commutators in this case can be bounded (see Appendix F) by

$$\left\|\mathrm{ad}^m_{\tilde{H}(t)}\left(\frac{\partial H(t)}{\partial t}\right)\right\| \le \Delta_q^m \left\|\frac{\partial H(t)}{\partial t}\right\|_{\mathrm{X}}, \tag{B41}$$

where $\Delta_q \equiv 2qM$. Note that compared to the previous three cases, the upper bound here does not contain the factorial $m!$, and hence we expect a tighter bound on the leakage. Following the same steps, we can obtain the recursive bounds for $g_k(t)$

$$g_k'(t) \le \sum_{m=1}^{k}\binom{k}{m} g_{k-m}(t)\Delta_q^{m-1}\lambda'(t)n. \tag{B42}$$

Similarly, we can define another polynomial $f_k^{(2)}(x)$ for comparison with $g_k(t)$. In this case, the recurrence relation for $f_k^{(2)}(x)$ becomes (c.f. Eq. (B34))

$$f_k^{(2)}(x) \equiv \int_0^x \mathrm{d}s \sum_{m=1}^{k} \binom{k}{m} f_{k-m}^{(2)}(s), \tag{B43}$$

where we set $f_0^{(2)}(x) = 1$ and $f_1^{(2)}(x) = x$ and we can verify that

$$g_k(t) \leq \Delta_q^k f_k^{(2)} \left(\frac{\lambda_t n}{\Delta_q} \right). \tag{B44}$$

One can use the generating function approach (or alternatively use the approach in Appendix B 5) to obtain the solution of the (B43)

$$f_k^{(2)}(x) = \sum_{j=0}^{k} \left\{ {k \atop j} \right\} x^j, \tag{B45}$$

where $\left\{ {k \atop j} \right\}$ is the Stirling number of the second kind. We can set $x = \frac{\lambda_t n}{\Delta_q}$ and use Markov's inequality to obtain

$$\epsilon_t(d)^2 \leq \min_k \frac{g_k(t)^2}{(dn)^{2k}} \leq \min_k \frac{f_k^{(2)}(x)^2}{(dn/\Delta_q)^{2k}}. \tag{B46}$$

Note that if $\hat{Y}$ is a random variable from the mean-x Poisson distribution, its kth moment is known to be exactly $f_k^{(2)}(x)$, i.e., $\mathbf{E}(\hat{Y}^k) = f_k^{(2)}(x)$ [11]. In addition, its moment-generating function

$$\mathbf{E}(e^{\tau \hat{Y}}) = \sum_{k=0}^{\infty} \frac{\tau^k}{k!} \mathbf{E}(\hat{Y}^k) = e^{x(e^\tau - 1)} \tag{B47}$$

also exists for any τ. A known result [12] on bounding the best k-moment bound by the Chernoff bound gives the following relation:

$$\min_k \frac{f_k^{(2)}(x)}{(dn/\Delta_q)^k} \leq \inf_{\tau \geq 0} \mathbf{E}(e^{\tau \hat{Y}}) e^{-\frac{dn}{\Delta_q}\tau}. \tag{B48}$$

Then we can obtain an explicit upper bound for $d > \lambda_t$:

$$\begin{aligned} \epsilon_t(d) &\leq \inf_{\tau \geq 0} \exp\left(x(e^\tau - 1) - \frac{dn}{\Delta_q}\tau \right) \\ &= \exp\left(-n \frac{\lambda_t}{\Delta_q} \left(\frac{d}{\lambda_t} \ln \frac{d}{\lambda_t} - (\frac{d}{\lambda_t} - 1) \right) \right). \end{aligned} \tag{B49}$$

3. Bounds for perturbations of general scalings

In previous sections we discussed cases with linear-scaling perturbations and energy windows, with bounded local norms. Here, we roughly estimate the leakage bounds in general settings where all parameters can scale differently with n.

In general, we can still define

$$\|H(t)\|_{\mathrm{loc}} \leq M, \tag{B50}$$

where M is a constant that may scale with n instead of an $O(1)$ constant previously, but still independent of t.

For a strictly q-local $H(t)$, we can define

$$\Lambda_t \equiv \int_0^t \left\| \frac{\partial H(\tau)}{\partial \tau} \right\|_{\mathrm{X}} \mathrm{d}\tau, \tag{B51}$$

where Λ_t can have generic scaling of n apart from the previous linear scaling. (Note we use the symbol Λ here, and in the previous cases, $\Lambda_t = \lambda(t)n$.) Since $\|H(t)\| \le nM$, we require that

$$\Lambda_t \lesssim O(nM), \tag{B52}$$

otherwise the change of the $H(t)$ will be too large to be called perturbations. One can also define $\Lambda_t \equiv \int_0^t \|\mathrm{d}H\|_{q\text{-X}}$ for quasi-q-local perturbations.

Similarly, we set the initial state $|\psi(0)\rangle$ as one eigenstate of $H(0)$ with energy E_0, and let the state $|\psi(t)\rangle$ evolve under $H(t)$. At time t, we define the eigenstates of $H(t)$ as $|\phi_j(t)\rangle$ with energy $E_j(t)$, and expand the evolving state in the instantaneous eigenbasis

$$|\psi(t)\rangle = \sum_j a_j(t)\, |\phi_j(t)\rangle\,. \tag{B53}$$

We set the energy window $D > \Lambda_t$ (usually the same scaling as Λ_t) accordingly and define the leakage $\Xi_t(D)$ as

$$\Xi_t(D) \equiv \sqrt{\sum_{j,|E_j(t)-E_0|\ge D} |a_j(t)|^2}. \tag{B54}$$

Following similar procedures, we can bound the cases whose nested commutators $\mathrm{ad}^m_{H(t)}(H'(t))$ increase factorially $O(m!\Delta_q^m)$ by $\Xi_t^{(1)}(D)$

$$\Xi^{(1)}_{\Lambda_t,\Delta_q}(D) \equiv \left(\frac{\Delta_q}{D}\right)^{k_D} \frac{\Gamma\left(\frac{\Lambda_t}{\Delta_q} + k_D\right)}{\Gamma\left(\frac{\Lambda_t}{\Delta_q}\right)}, \tag{B55}$$

where $\Delta_q \equiv 2qM$ and $k_D \equiv \left\lceil \frac{D-\Lambda_t}{\Delta_q} \right\rceil$ is well defined only for $D > \Lambda_t$. This can be compared to Eq. (B38) in the previous case.

Similarly, for $H(t)$ whose $\mathrm{ad}^m_{H(t)}(H'(t))$ scales exponentially $O(\Delta_q^m)$, the leakage can be bounded by

$$\Xi^{(2)}_{\Lambda_t,\Delta_q}(D) \equiv \exp\left(-\frac{\Lambda_t}{\Delta_q}\left(\frac{D}{\Lambda_t}\ln\frac{D}{\Lambda_t} - (\frac{D}{\Lambda_t} - 1)\right)\right), \tag{B56}$$

which can be compared to Eq. (B49).

If we set $D/\Lambda_t > 1$ to be a $\Theta(1)$ constant independent of t and Λ_t is large, one can verify that both bounds scale the same as

$$\Xi_t(D) \lesssim \exp\left(-\Omega(\frac{\Lambda_t}{2qM})\right). \tag{B57}$$

Specifically, for $H(t)$ with a $\Theta(1)$ local norm M and a general scaling $\Lambda_t \sim \Theta(f(n))$, the bound is roughly $e^{-\Omega(f(n))}$.

4. Properties of $f_k^{(1)}$

In this section, we prove that the $f_k^{(1)}(x)$'s we found

$$f_n^{(1)}(x) = \prod_{m=0}^{n-1}(x+m) = \sum_{j=0}^{n} \begin{bmatrix} n \\ j \end{bmatrix} x^j \tag{B58}$$

indeed satisfies the recurrence relation (B34), where $\begin{bmatrix} n \\ j \end{bmatrix}$ is the unsigned Stirling number of the first kind by definition. The $\begin{bmatrix} n \\ j \end{bmatrix}$ also has a combinatorial interpretation: $\begin{bmatrix} n \\ j \end{bmatrix}$ counts the number of permutations when dividing the n elements into j disjoint cycles. It is easy to verify that $f_0^{(1)}(x)$ and $f_1^{(1)}(x)$ satisfy the relation (B34). Suppose that Eq. (B58)

holds for all $n < k$. At the k-th step, we can obtain $f_k^{(1)}(x)$ from the recurrence relation

$$\begin{aligned} f_k^{(1)}(x) &= \sum_{m=1}^{k} \binom{k}{m} (m-1)! \sum_{j=0}^{k-m} \left[{k-m \atop j} \right] \frac{x^{j+1}}{j+1} \\ &= \sum_{j=0}^{k-1} \frac{x^{j+1}}{j+1} \sum_{m=1}^{k-j} \binom{k}{m} (m-1)! \left[{k-m \atop j} \right]. \end{aligned} \tag{B59}$$

We are going to prove the following identity combinatorially:

$$\frac{1}{j+1} \sum_{m=1}^{k} \binom{k}{m} (m-1)! \left[{k-m \atop j} \right] = \left[{k \atop j+1} \right]. \tag{B60}$$

The RHS can be easily interpreted as the number of permutations in dividing k elements into $j+1$ disjoint cycles. The LHS can be interpreted as follows: 1. Choose a cycle C of m elements from the total k elements, which gives $\binom{k}{m}$ choices. The cycle C has $(m-1)!$ permutations. 2. Dividing the rest of elements into j cycles, which gives $\left[{k-m \atop j} \right]$ permutations. 3. Sum over all possible m, we get the number of permutations in dividing k elements into $j+1$ disjoint cycles. As any of the $j+1$ cycles can be labeled as C, we need an additional factor $1/(j+1)$ for multiple counting. Therefore, the identity is proved. Using $\left[{k \atop 0} \right] = 0$ and $\left[{i \atop j} \right] = 0$ for any $i < j$, we obtain

$$f_k^{(1)}(x) = \sum_{j=0}^{k-1} \left[{k \atop j+1} \right] x^{j+1} = \sum_{j=0}^{k} \left[{k \atop j} \right] x^j, \tag{B61}$$

which is exactly Eq. (B58).

5. Properties of $f_k^{(2)}$

In this section, we want to confirm that

$$f_n^{(2)}(x) = \sum_{j=0}^{n} \left\{ {n \atop j} \right\} x^j \tag{B62}$$

satisfies the recurrence relation (B43), where $\left\{ {n \atop j} \right\}$ is the Stirling number of the second kind. The $\left\{ {n \atop j} \right\}$ has a combinatorial interpretation: $\left\{ {n \atop j} \right\}$ counts the number of ways to partition n elements into j unlabeled non-empty sets. It is easy to verify that $f_0^{(2)}(x) = 1$ and $f_1^{(2)}(x) = x$ satisfy the relation (B43). Suppose that Eq. (B62) holds for all $n < k$. In the k-th step, we can obtain f_k from the recurrence relation.

$$\begin{aligned} f_k^{(2)}(x) &= \sum_{m=1}^{k} \binom{k}{m} \sum_{j=0}^{k-m} \left\{ {k-m \atop j} \right\} \frac{x^{j+1}}{j+1} \\ &= \sum_{j=0}^{k-1} \frac{x^{j+1}}{j+1} \sum_{m=1}^{k-j} \binom{k}{m} \left\{ {k-m \atop j} \right\}. \end{aligned} \tag{B63}$$

We are going to prove the following identity combinatorially:

$$\frac{1}{j+1} \sum_{m=1}^{k} \binom{k}{m} \left\{ {k-m \atop j} \right\} = \left\{ {k \atop j+1} \right\}. \tag{B64}$$

The RHS can be easily interpreted as the number of partitions in dividing k elements into $j+1$ non-empty sets. The LHS can be interpreted as follows: 1. Choose a set C containing m elements from the total k elements, which gives $\binom{k}{m}$ choices. 2. Divide the rest of the elements into j non-empty sets, which gives $\left\{ {k-m \atop j} \right\}$ ways. 3. Sum over all

Perturbation type	Dynamical localization		Eigenstate localization	Slow mixing of Gibbs sampler
	Exponential time	Infinite time		
Static strictly q-local	λ (C21), ξ (C20)	λ (C44), ξ (C43)	λ (D17), ξ (D16)	λ (E16), ξ (E15)
Static quasi-$q_\star$-local	λ (C30), ξ (C29)	$\lambda, q_\star$ (C56), ξ (C55)	$\lambda, q_\star$ (D29), ξ (D28)	$\lambda, q_\star$ (E22), ξ (E21)
Time-dependent (quasi-)q-local	(C21), (C20); (C30), (C29)	-	-	-

TABLE S1. Summary of requirements for perturbation strength parameters λ & $q_\star$ and leakage bounds $e^{-\xi n}$ for different stabilities. For strictly q-local perturbations, the strength is depicted by λ. For quasi-$q_\star$-local perturbations, the strength is depicted by both λ and $q_\star$. In closed systems, these parameters are mainly determined by the energy barrier height bn and the initial energy density ε_0. In open systems, they are also determined by the inverse temperature β.

possible m, we get the number of partitions in dividing k elements into $j+1$ nonempty sets. As any of the $j+1$ sets can be labeled as C, we need an additional factor $1/(j+1)$ for multiple counting. Therefore, the identity is proved. Using $\left\{ {k \atop 0} \right\} = 0$ and $\left\{ {i \atop j} \right\} = 0$ for any $i < j$, we obtain

$$f_k^{(2)}(x) = \sum_{j=0}^{k-1} \left\{ {k \atop j+1} \right\} x^{j+1} = \sum_{j=0}^{k} \left\{ {k \atop j} \right\} x^j, \tag{B65}$$

which is exactly Eq. (B62).

Appendix C: Proof of the dynamical localization

We consider a state $|\psi(t)\rangle$ initialized at eigenstate of H_C inside w_0 with energy $E_0 = \varepsilon_0 n + E_g$, evolving under $H(t) = H_C + V(t)$. We assume H_C is a good c/qLDPC code with linear soundness and satisfies the property discussed in Sec. II. An essential property from the low-density requirement is that H_C is at most q_C-local and

$$\|H_C\|_{\text{loc}} \le p_C, \tag{C1}$$

where p_C is the maximum number of checks involving one single qubit and q_C is the maximum qubit number in one check.

We are going to prove that $|\psi(t)\rangle$ remains inside w_0 up to an exponentially/infinitely long time under different conditions, with the leakage being exponentially small. The dynamical localization is defined in the eigenbasis of H_C. For eigenstates $|\phi_j\rangle$ of H_C, we denote $P_w \equiv \sum_{\phi_j \in w} |\phi_j\rangle \langle\phi_j|$ as the projector to the cluster w and $P_W = \sum_w P_w$ as the projector to all clusters. But there are also high-energy states not in any cluster, and we denote the projector to these high-energy states as $P_> \equiv 1 - P_W$, i.e.,

$$P_> = 1 - \sum_w P_w. \tag{C2}$$

Since all eigenstates of H_C below $E_B \equiv E_g + bn$ are divided into clusters w's, $P_>$ projects a state to its components with energies higher than E_B.

As different clusters are globally separated, a state cannot transit to other clusters directly without first overcoming the energy barriers. Thus, an essential step in proving dynamical localization is to bound the total weight of high-energy eigenstates of H_C at arbitrary time t. To bound the high-energy weights at time t_1, we can invoke Theorem 1 for an extended evolution under the following segments of the Hamiltonian

$$H_{\text{ext}}(t) = \begin{cases} H_C & \text{for } t = 0^-, \\ H_C + V(t) & \text{for } 0 \le t \le t_1, \\ H_C & \text{for } t = t_1^+, \end{cases} \tag{C3}$$

where $H_{\text{ext}}(t)$ at $t = 0^-, 0$ and t_1, t_1^+ are linearly connected (similar to Eq. (10)) and we let the system evolve from 0^- to t_1^+. From Schrödinger's equation, we know that $|\psi(t_1^+)\rangle = |\psi(t_1)\rangle$, and thus we can bound the high-energy leakage of $|\psi(t_1)\rangle$ in the eigenbasis of H_C at any time t_1. This results in the following lemma.

Lemma C.1. We consider the $|\psi(t)\rangle$ evolving under $H(t) = H_C + V(t)$ defined above with strictly q-local $V(t)$, where we assume $q \geq q_C$ for simplicity. For Case 1 and Case 2, we can bound the high-energy contribution in the eigenbasis of H_C by

$$\|P_> |\psi(t)\rangle\| \leq \epsilon^{(1)}_{2\lambda_t,\Delta_q}(b - \varepsilon_0), \tag{C4}$$

For Case 3, we have

$$\|P_> |\psi(t)\rangle\| \leq \epsilon^{(2)}_{2\lambda_t,\Delta_q}(b - \varepsilon_0), \tag{C5}$$

where $\lambda_t \equiv \frac{1}{n}\int_{0^-}^{t} \|H'_{\text{ext}}(\tau)\|_{\text{X}}\, \mathrm{d}\tau$ and $\Delta_q = 2qM$. If $V(t)$ is quasi-$q_\star$-local and $H(t)$ satisfies Case 4, we have

$$\|P_> |\psi(t)\rangle\| \leq \epsilon^{(1)}_{2\lambda_t,\Delta_{q_\star}}(b - \varepsilon_0), \tag{C6}$$

where $\lambda_t \equiv \frac{1}{n}\int_{0^-}^{t} \|H'_{\text{ext}}(\tau)\|_{q_\star\text{-X}}\, \mathrm{d}\tau$ and $\Delta_{q_\star} = 2q_\star M$. In all cases we only require $\lambda_t < (b-\varepsilon_0)/2$, or equivalently, $\varepsilon_0 < b - 2\lambda_t$.

Proof. At a given time $t = t_1$, we consider $|\psi(t_1^+)\rangle$ from the extended evolution $H_{\text{ext}}(t)$ in (C3), which produces exactly the same state as $|\psi(t_1)\rangle$ under $H(t) = H_C + V(t)$ from $t = 0$ to t_1. For the evolution from t_1 to t_1^+, the Hamiltonian is effectively $H_{\text{ext}}(t) = H_C + (1 - \frac{t-t_1}{\tau})V(t_1)$ for $t \in [t_1, t_1 + \tau]$ with $\tau \to 0^+$. The evolution of the state and the Hamiltonian is then

$$\begin{aligned} H_C &\xrightarrow{t\in[0^-,0]} H(0) \xrightarrow{t\in[0,t_1]} H(t_1) \xrightarrow{t\in[t_1,t_1^+]} H_C, \\ |\psi(0)\rangle &\xrightarrow{t\in[0^-,0]} |\psi(0)\rangle \xrightarrow{t\in[0,t_1]} |\psi(t_1)\rangle \xrightarrow{t\in[t_1,t_1^+]} |\psi(t_1)\rangle\,. \end{aligned} \tag{C7}$$

We then use Theorem 1 to bound the high-energy components in the instantaneous eigenbasis of $H_{\text{ext}}(t_1^+)$ from from $t = 0^-$ to t_1^+. By construction, now the instantaneous Hamiltonian $H_{\text{ext}}(t_1^+)$ is exactly the original Hamiltonian

$$H_{\text{ext}}(t_1^+) = H_C. \tag{C8}$$

Therefore, the high-energy component $\|P_> |\psi(t_1)\rangle\|$ in the eigenbasis of H_C at t_1 can be directly bounded by Theorem 1 after the extension.

With such extension of evolution, the total variation here increases by the additional virtual evolution from $H(t_1) = H_C + V(t_1)$ back to H_C from t_1 to t_1^+. The contribution to the total variation is exactly $\|V(t_1)\|_{\text{X}}$. We can use

$$\int_{t_1}^{t_1^+} \|\mathrm{d}H_{\text{ext}}\|_{\text{X}} = \|V(t_1)\|_{\text{X}} = \left\|\int_{0^-}^{t_1} \mathrm{d}H_{\text{ext}}\right\|_{\text{X}} \leq \int_{0^-}^{t_1} \|\mathrm{d}H_{\text{ext}}\|_{\text{X}} \equiv \lambda_{t_1} n \tag{C9}$$

to bound the variation by $\lambda_{t_1} n$. Note that we have re-defined the total variation as $\int_{0^-}^{t_1} \|\mathrm{d}H_{\text{ext}}\|_{\text{X}}$ instead of $\int_{0}^{t_1} \|\mathrm{d}H\|_{\text{X}}$. Thus, we can bound the total variation for the extended evolution by $2\lambda_{t_1} n$, at a cost of a factor of two,

$$\int_{0^-}^{t_1^+} \|H'_{\text{ext}}(t)\|_{\text{X}} \mathrm{d}t \leq 2\lambda_{t_1} n. \tag{C10}$$

Since the procedure works for arbitrary t_1, by setting

$$d = b - \varepsilon_0,\ \ \lambda = 2\lambda_t,\ \ \Delta = \Delta_q \equiv 2qM, \tag{C11}$$

in Theorem 1 for Case 1 - 4, we obtain the all the bounds respectively. □

We remark that for Cases 2 - 4, the condition $[H'(t), V(t)] = 0$ is reduced to $[V'(t), V(t)] = 0$, which can be satisfied for perturbations such as $V(t) = f(t)V_0$. In the following, we show the detailed proof for the localization time by using the above lemma that we just proved.

1. Exponentially long time dynamical localization

In this section, we consider the evolution under the following time-dependent Hamiltonian:

$$H(t) = H_C + V(t), \tag{C12}$$

where $V(t)$ is a time-dependent perturbation. We require that $V(t)$ satisfies $\int_{0^-}^{T} \|H'_{\rm ext}(t)\|_{\rm X} \le \lambda n$ for strictly q-local cases. This is equivalent to

$$\|V(0)\|_{\rm X} + \int_0^T \|V'(t)\|_{\rm X} \le \lambda n. \tag{C13}$$

Similarly, for quasi-$q_\star$-local $V(t)$ in Case 4, we require that

$$\|V(0)\|_{q\text{-X}} + \int_0^T \|V'(t)\|_{q\text{-X}} \le \lambda n. \tag{C14}$$

a. *Strictly q-local perturbation*

Here, we are going to prove the dynamical localization for Case 3, and the results for Case 1 and 2 can be obtained by replacing $\epsilon^{(2)}$ with $\epsilon^{(1)}$.

In condition $q < \nu_2$, we have $P_w V(t) P_{w'} = 0$ for $w \neq w'$, i.e., there are no transition elements between two different clusters. Consider the expectation of P_{w_0} for an initial eigenstate with energy $\varepsilon_0 n + E_g$ inside the cluster w_0. Its derivative is given by

$$\frac{\mathrm{d}}{\mathrm{d}t} \langle \psi(t)| P_{w_0} |\psi(t)\rangle = i \left\langle [H(t), P_{w_0}] \right\rangle_t. \tag{C15}$$

Note that

$$\begin{aligned} [H(t), P_{w_0}] &= [V(t), P_{w_0}] \\ &= (1 - P_{w_0}) V(t) P_{w_0} - P_{w_0} V(t)(1 - P_{w_0}) \\ &= P_> V(t) P_{w_0} - P_{w_0} V(t) P_>, \end{aligned} \tag{C16}$$

where we have used $P_{w'} V(t) P_w = 0$ for $w \neq w'$ and $P_> = 1 - \sum_w P_w$ in the second and third lines above. We have already proved in Lemma. C.1 that $\|P_> |\psi(t)\rangle\|$ is exponentially small

$$\|P_> |\psi(t)\rangle\| \le \epsilon^{(2)}_{2\lambda_t, \Delta_q}(b - \varepsilon_0). \tag{C17}$$

Thus, we can bound the derivative

$$\left| \frac{\mathrm{d}}{\mathrm{d}t} \langle P_{w_0} \rangle_t \right| \le 2 \|P_> |\psi(t)\rangle\| \cdot \|V(t) P_{w_0} |\psi(t)\rangle\| \le 2\lambda_t n \epsilon^{(2)}_{2\lambda_t, \Delta_q}(b - \varepsilon_0). \tag{C18}$$

Using $\langle P_{w_0} \rangle_{t=0} = 1$ and $\lambda_t \le \lambda_T \equiv \lambda$, we can obtain

$$\langle 1 - P_{w_0} \rangle_T \le 2\lambda n T \epsilon^{(2)}_{2\lambda, \Delta_q}(b - \varepsilon_0). \tag{C19}$$

Since $\epsilon^{(2)}_{2\lambda, \Delta_q}(b - \varepsilon_0) \le e^{-\xi n}$, where

$$\xi = \frac{2\lambda}{\Delta_q} \left(\frac{b - \varepsilon_0}{2\lambda} \ln \frac{b - \varepsilon_0}{2\lambda} - \left(\frac{b - \varepsilon_0}{2\lambda} - 1\right) \right), \tag{C20}$$

the leakage outside w_0 is exponentially small as long as $T \le \frac{1}{2\lambda n} e^{\xi_1 n}$ with any $\xi_1 < \xi$.

Therefore, any initial eigenstate of H_C localized in w_0, with energy $E_g + \varepsilon_0 n$ with $\varepsilon_0 < b$, remains localized inside w_0 under q-local time-dependent perturbation $V(t)$ up to exponentially long time $T < \frac{1}{2\lambda n} e^{\xi_1 n}$, as long as the perturbation strength satisfies

$$\lambda < \frac{b - \varepsilon_0}{2} \equiv \lambda_{\rm Exp}, \tag{C21}$$

with leakage outside w_0 bounded by $e^{-(\xi - \xi_1)n}$. Here, one can choose any ξ_1 such that $0 < \xi_1 < \xi$ to fulfill the exponentially long dynamical localization.

b. Quasi-$q_\star$-local perturbation

Here, we are going to prove the dynamical localization for Case 4. As a quasi-q-local perturbation contains terms that can be very non-local, thus the trick of zero matrix elements between two different clusters does not work. We have to proceed with a slight modification. For $H(t) = H_C + V(t)$ with quasi-$q_\star$-local V, we define

$$H(t) = H_C + V_L(t) + V_R(t), \tag{C22}$$

where $V(t)$ is divided into the local part $V_L(t)$ and the remainder $V_R(t)$. $V_L(t)$ includes all terms in $V(t)$ with the k-locality satisfying $k < \nu_2$ with $\nu_2 \sim O(n)$, so that they do not introduce direct matrix elements between different clusters, and the remainder $V_R(t) \equiv V(t) - V_L(t)$ contains all the non-local terms in $V(t)$. Then the local part $V_L(t)$ satisfies

$$P_{w'} V_L(t) P_w = 0, \text{ for } \; w \neq w'. \tag{C23}$$

By $\int_{0^-}^t \|H'(\tau)\|_{q_\star\text{-X}} \mathrm{d}\tau \le \lambda_t n$, we have

$$\|V_R(t)\| \le \lambda_t n e^{-\nu_2/q_\star}. \tag{C24}$$

Similarly, we have

$$\frac{\mathrm{d}}{\mathrm{d}t} \langle \psi(t)| P_{w_0} |\psi(t)\rangle = i \left\langle [H(t), P_{w_0}] \right\rangle_t . \tag{C25}$$

We can rewrite the r.h.s. by noting that

$$[H(t), P_{w_0}] = [V_L(t) + V_R(t), P_{w_0}] = P_> V_L(t) P_{w_0} - P_{w_0} V_L(t) P_> + [V_R(t), P_{w_0}]. \tag{C26}$$

Using Lemma C.1, we can bound the derivative by

$$\left| \frac{\mathrm{d}}{\mathrm{d}t} \langle P_{w_0} \rangle_t \right| \le 2\lambda_t n \left(\epsilon^{(1)}_{2\lambda_t, \Delta_{q_\star}}(b - \varepsilon_0) + e^{-\nu_2/q_\star} \right). \tag{C27}$$

Using $\langle P_{w_0} \rangle_{t=0} = 1$ and $\lambda_t \le \lambda_T \equiv \lambda$, we obtain

$$\langle 1 - P_{w_0} \rangle_T \le 2\lambda n T \left(\epsilon^{(1)}_{2\lambda, \Delta_{q_\star}}(b - \varepsilon_0) + e^{-\nu_2/q_\star} \right). \tag{C28}$$

Here $\nu_2 = d_C - 2\gamma n \sim O(n)$ by definition. Since both $\epsilon^{(1)}_{2\lambda, \Delta_{q_\star}}(b - \varepsilon_0)$ and $e^{-\nu_2/q_\star}$ decay exponentially with n, we can define

$$\xi = \min\left(\frac{2\lambda}{\Delta_{q_\star}} \Big(\frac{b - \varepsilon_0}{2\lambda} - 1 - \ln \frac{b - \varepsilon_0}{2\lambda} \Big), \frac{\nu_2}{q_\star n} \right). \tag{C29}$$

Then the leakage outside w_0 is exponentially small as long as $T \le \frac{1}{2\lambda n} e^{\xi_1 n}$ with any $\xi_1 < \xi$.

Therefore, any initial eigenstate of H_C localized in w_0, with energy $E_g + \varepsilon_0 n$ with $\varepsilon_0 < b$, remains localized inside w_0 under quasi-$q_\star$-local time-dependent perturbation $V(t)$ up to exponentially long time $T < e^{\xi_1 n}$, as long as the perturbation strength satisfies

$$\lambda < \frac{b - \varepsilon_0}{2} \equiv \lambda_{\mathrm{Exp}}, \tag{C30}$$

with leakage outside w_0 bounded by $e^{-(\xi - \xi_1)n}$. Here, one can choose any ξ_1 such that $0 < \xi_1 < \xi$. We note that the requirement for λ is the same as that for q-local perturbation, but the requirement for ξ_1 is different.

2. Infinitely long time dynamical localization in cLDPC codes

In this section, we are going to show that the dynamical localization can be infinitely long in some classical models with linear energy barriers.

We consider the following static Hamiltonian (similarly defined in [1])

$$H = H_C + V_0 + H_{\mathrm{d}}, \tag{C31}$$

where H_C is a good cLDPC code with linear soundness and H_{d} is a small diagonal detuning defined by

$$H_{\mathrm{d}} \equiv \frac{1}{n^2}\sum_j h_j^z Z_j, \tag{C32}$$

where h_j^z can be independently randomly chosen from the Gaussian distribution with mean 0 and variance 1. We note that H_{d} only contributes $O(\frac{1}{n})$ to the total variation of H from H_C, so it is negligible when considering leakage bounds in the energy space.

While the setup of H here is only a special case in the exponentially-long-time dynamical localization discussed in Appendix C 1, it remains nontrivial to prove that the localization time can be truly infinite at finite n.

In Appendix D, we prove that if $\lambda \sim O(1)$ is small enough (also $q_\star \sim O(1)$ is small enough for quasi-$q_\star$-local perturbations), the Hamiltonian H exhibits eigenstate localization for $E' < E_g + bn$ (see explicit bounds for λ in Eq. (D3) and (D4)). Here, the bound for λ is a function of $E_B - E'$ and will vanish if $E_B - E' \to 0$

Suppose we have an initial state $|\psi(t)\rangle$ initialized at eigenstate of H_C inside w_0 with energy $E_0 = \varepsilon_0 n + E_g$, evolving under $H = H_C + V_0 + H_{\mathrm{d}}$. From Theorem 2 we know that $|\psi(0)\rangle$ roughly is exponentially localized in the energy window $[E_0 - \lambda n, E_0 + \lambda n]$ in the spectrum of H. From Appendix D, we know that any eigenstate of H below $E_B \equiv E_g + bn$ is localized in one single cluster if λ is sufficiently small. Thus, as long as the majority of $|\psi(0)\rangle$ comes from the localized eigenstates inside w_0, we can bound the leakage for all time. We show the detailed proof for this argument below.

a. *Strictly q-local perturbation*

In this subsection, we assume $V_0 + H_{\mathrm{d}}$ is strictly q-local and

$$\|V_0 + H_{\mathrm{d}}\|_{\mathrm{X}} \le \lambda n. \tag{C33}$$

To rigorously demonstrate our arguments at the beginning of the section, we can expand the $|\psi(0)\rangle$, an eigenstate of H_C with energy $E_g + \varepsilon_0 n$, in the eigenbasis of H

$$\begin{aligned}|\psi(0)\rangle &= \sum_j a_{\omega_0,j}|\phi_{\omega_0,j}\rangle + \sum_{\omega\neq\omega_0,j} a_{\omega,j}|\phi_{\omega,j}\rangle + |\psi_R\rangle \\ &\equiv |\psi_{w_0}\rangle + |\psi_{\neq w_0}\rangle + |\psi_R\rangle,\end{aligned} \tag{C34}$$

where the first two terms contain all localized eigenstates (see Appendix D for details) with energy below $E_g + (\varepsilon_0 + d)n$ (with $d > \lambda$) of H and $|\psi_R\rangle$ is the remainder. The notion $|\phi_{\omega_0,j}\rangle$ denotes that it is localized inside w_0 with exponentially small leakage, which is proved in Appendix D. The eigenstate localization condition requires that $\varepsilon_0 + d < b$. From Theorem 2 we know that the leakage is

$$\|\,|\psi_R\rangle\,\| \le \epsilon^{(2)}_{\lambda,\Delta_q}(d). \tag{C35}$$

From Eq. (D15) we know that for energy below $E_g + (\varepsilon_0 + d)n$, all eigenstates of H is localized when δ_W is sufficiently large, which is satisfied with high probability $1 - e^{-2(1-\ln 2)n}$ and is shown in Appendix D. The leakage outside w_0 can be bounded by

$$\|(1 - P_w)|\phi_{w,j}\rangle\| \le e^{-\xi(\varepsilon_0+d,\lambda)n}, \tag{C36}$$

where we can set $\varepsilon' = \varepsilon_0 + d$ and

$$\xi(\varepsilon',\lambda) \equiv \frac{b-\varepsilon'}{\Delta_q}\ln\frac{b-\varepsilon'}{\lambda} - \frac{b-\varepsilon'-\lambda}{\Delta_q} - 2. \tag{C37}$$

Thus, the contribution to w_0 in $|\psi_{\neq w_0}\rangle$ of Eq. (C34) can be bounded by

$$\left\| P_{w_0}\sum_{\omega\neq\omega_0,j} a_{\omega,j}|\phi_{\omega,j}\rangle\right\| \le 2^{n/2}e^{-\xi(\varepsilon',\lambda)n}. \tag{C38}$$

Thus, contribution of the $|\psi_{w_0}\rangle$ of Eq. (C34) cannot be too small

$$\begin{aligned}
&\| |\psi_{\omega_0}\rangle \| \geq \|P_{\omega_0} |\psi_{\omega_0}\rangle \| \\
&\geq \|P_{\omega_0} |\psi(0)\rangle \| - \|P_{\omega_0} |\psi_{\neq w_0}\rangle \| - \|P_{\omega_0} |\psi_R\rangle \| \\
&\geq 1 - e^{-(\xi-\frac{1}{2}\ln 2)n} - \epsilon^{(2)}_{\lambda,\Delta_q}(d) \equiv 1 - \epsilon_{\neq\omega_0},
\end{aligned} \tag{C39}$$

where we have defined

$$\epsilon_{\neq\omega_0} \equiv e^{-(\xi-\frac{1}{2}\ln 2)n} + \epsilon^{(2)}_{\lambda,\Delta_q}(d). \tag{C40}$$

We can also bound $\| |\psi_{\neq\omega_0}\rangle \|$ by

$$\| |\psi_{\neq\omega_0}\rangle \| \leq \sqrt{1 - \| |\psi_{\omega_0}\rangle \|^2} \leq \sqrt{2\epsilon_{\neq\omega_0}}. \tag{C41}$$

Since the evolution under H will not change the weights on its eigenstates, $\| |\psi_{\omega_0}\rangle \|$ and $\| |\psi_{\neq\omega_0}\rangle \|$ are conserved at all times. Then we can bound the leakage of $|\psi(t)\rangle$ outside w_0 at any t by

$$\begin{aligned}
&\|(1 - P_{w_0}) |\psi(t)\rangle \| = \|(1 - P_{w_0}) e^{-iHt} |\psi(0)\rangle \| \\
&\leq \|(1 - P_{w_0}) |\psi_{\omega_0}\rangle \| + \| |\psi_{\neq\omega_0}\rangle \| + \| |\psi_R\rangle \| \\
&\leq 2\sqrt{2\epsilon_{\neq\omega_0}} + \epsilon^{(2)}_{\lambda,\Delta_q}(d).
\end{aligned} \tag{C42}$$

For any $d > \lambda$, the $\epsilon^{(2)}_{\lambda,\Delta_q}(d)$ is exponentially small. Thus, we only need to ensure that $e^{-(\xi-\frac{1}{2}\ln 2)n}$ is exponentially small, i.e., $\xi > \frac{1}{2}\ln 2$. For convenience, we define $\xi_\infty \equiv (\xi - \frac{1}{2}\ln 2)/2$ and set $d = (b - \varepsilon_0)/2 > \lambda$, and obtain

$$\xi_\infty = \frac{b - \varepsilon_0}{4\Delta_q} \ln \frac{b - \varepsilon_0}{2\lambda} - \frac{b - \varepsilon_0 - 2\lambda}{4\Delta_q} - 1 - \frac{1}{4}\ln 2. \tag{C43}$$

One can verify that if

$$\lambda < \frac{b - \varepsilon_0}{2e} e^{-\frac{2e\Delta_q}{b-\varepsilon_0}} \equiv \lambda_\infty, \tag{C44}$$

we can ensure that $\xi_\infty > 0$.

Thus, if λ satisfies the above condition, any initial eigenstate of H_C localized in w_0, with energy $E_g + \varepsilon_0 n$ with $\varepsilon_0 < b$, remains localized inside w_0 up to infinite time. The leakage outside w_0 can be bounded by

$$\|(1 - P_{w_0}) |\psi(t)\rangle \| \leq e^{-\xi_\infty n}. \tag{C45}$$

If we can compare the requirement for λ in Eq. (C44) with exponentially long dynamical localization (C30) and eigenstate localization (D17), we can find that

$$\lambda_\infty < \lambda_{\text{Eigen}} < \lambda_{\text{Exp}}. \tag{C46}$$

Thus, the requirement for infinite-time dynamical localization is the most stringent.

b. *Quasi-$q_\star$-local perturbation*

In this subsection, we assume $V_0 + H_\text{d}$ is quasi-$q_\star$-local and

$$\|V_0 + H_\text{d}\|_{q_\star\text{-X}} \leq \lambda n. \tag{C47}$$

Similar to Eq. (C34), we can divide the initial state into three parts

$$|\psi(0)\rangle \equiv |\psi_{w_0}\rangle + |\psi_{\neq w_0}\rangle + |\psi_R\rangle, \tag{C48}$$

where the first two terms contain all localized eigenstates with energy below $E_g + (\varepsilon_0 + d)n$ (with $d > \lambda$) of H and $|\psi_R\rangle$ is the remainder. The eigenstate localization condition requires that $\varepsilon_0 + d < b$. From Theorem 2 we know that the leakage is

$$\| |\psi_R\rangle \| \leq \epsilon^{(1)}_{\lambda,\Delta_{q_\star}}(d). \tag{C49}$$

From Eq. (D27) we know that for energy below $E_g + (\varepsilon_0 + d)n$, all eigenstates of H is localized with high probability $1 - e^{-2(1-\ln 2)n}$, with leakage

$$\|(1 - P_w)\,|\phi_{w,j}\rangle\,\| \le e^{-\xi n}, \tag{C50}$$

where we can set $\varepsilon' = \varepsilon_0 + d$ and

$$\xi \equiv \min\left(\frac{b - \varepsilon' - \lambda}{\Delta_{q_\star}} - \frac{\lambda}{\Delta_{q_\star}} \ln \frac{b - \varepsilon'}{\lambda}, \frac{\nu_2}{nq_\star}\right) - 2. \tag{C51}$$

Similarly, we can bound the first two terms in Eq. (C48) by

$$\left\| P_{w_0} \sum_{\omega \neq \omega_0, j} a_{\omega,j}\,|\phi_{\omega,j}\rangle \right\| \le 2^{n/2} e^{-\xi n}, \tag{C52}$$

and

$$\|\,|\psi_{\omega_0}\rangle\,\| \ge \|P_{\omega_0}\,|\psi_{\omega_0}\rangle\,\| \ge 1 - e^{-(\xi - \frac{1}{2}\ln 2)n} - \epsilon^{(1)}_{\lambda,\Delta_{q_\star}}(d) \equiv 1 - \epsilon_{\neq\omega_0}. \tag{C53}$$

Then we can bound the leakage of $|\psi(t)\rangle$ outside w_0 at any t by

$$\|(1 - P_{w_0})\,|\psi(t)\rangle\,\| \le 2\sqrt{2\epsilon_{\neq\omega_0}} + \epsilon^{(1)}_{\lambda,\Delta_{q_\star}}(d). \tag{C54}$$

Similarly, we define $\xi_\infty \equiv (\xi - \frac{1}{2}\ln 2)/2$ and set $d = (b - \varepsilon_0)/2 > \lambda$, and only need to ensure that $\xi_\infty > 0$, where

$$\xi_\infty \equiv \min\left(\frac{b - \varepsilon_0 - 2\lambda}{4\Delta_{q_\star}} - \frac{\lambda}{2\Delta_{q_\star}} \ln \frac{b - \varepsilon_0}{2\lambda}, \frac{\nu_2}{2nq_\star}\right) - 1 - \frac{1}{4}\ln 2. \tag{C55}$$

One can verify that if

$$\lambda < \frac{b - \varepsilon_0}{2e},\; q_\star \le \min\left(\frac{(b - \varepsilon_0)(1 - 2/e)}{4eM}, \frac{\nu_2}{en}\right), \tag{C56}$$

then we can ensure that $\xi_\infty > 0$.

Thus, if λ and $q_\star$ satisfy the above condition, any initial eigenstate of H_C localized in w_0, with energy $E_g + \varepsilon_0 n$ with $\varepsilon_0 < b$, remains localized inside w_0 to infinite time. The leakage outside w_0 can be bounded by

$$\|(1 - P_{w_0})\,|\psi(t)\rangle\,\| \le e^{-\xi_\infty n}. \tag{C57}$$

Appendix D: Proof of the eigenstate localization

In this section, we consider the following Hamiltonian (similarly defined in [1])

$$H = H_C + V_0 + H_{\mathrm{d}}, \tag{D1}$$

where H_C is a good cLDPC code and $\|V_0\| \sim \lambda n$ is an arbitrary (quasi-)q-local perturbation. H_{d} is a small diagonal detuning defined by

$$H_{\mathrm{d}} \equiv \frac{1}{n^2} \sum_j h_j^z Z_j, \tag{D2}$$

where h_j^z can be independently randomly chosen from the Gaussian distribution with mean 0 and variance 1. We note that H_{d} only contributes $O(\frac{1}{n})$ to the total variation of H from H_C, so it is negligible when considering leakage bounds in the energy space. In addition, H_{d} will be infinitesimal in the thermodynamic limit. However, the polynomially small H_{d} is sufficient to lift the degeneracies in H_C to gaps above an exponentially small threshold. We remark that the essential ingredient here is not the specific form of H_{d}, but the fact that the degeneracies in cLDPC codes can be easily lifted by local detuning. In contrast, the ground state degeneracies of good qLDPC codes cannot be lifted

(with maximum separating $\sim \lambda^{O(n)}$) by any local perturbations [13, 14], similar to the property in topological order. We will see shortly that lifting the degeneracies is essential for eigenstate localization.

We are going to prove that the perturbed eigenstate $|\psi\rangle$ of H with energy $E' \equiv \varepsilon' n + E_g$ (with $\varepsilon' < b$) is exponentially localized in one cluster $w_\star$. For strictly q-local V_0, we require that $\|V_0\|_{\mathrm{X}} \leq \lambda n$ and

$$\lambda < \frac{b-\varepsilon'}{e}\exp\left(-\frac{2\Delta_q}{b-\varepsilon'}\right). \tag{D3}$$

For quasi-$q_\star$-local V_0, we require that $\|V_0\|_{q_\star\text{-X}} \leq \lambda n$ and

$$\lambda < \frac{b-\varepsilon'}{e},\ q_\star \leq \min\left(\frac{(b-\varepsilon')(1-2/e)}{4M}, \frac{\nu_2}{2n}\right). \tag{D4}$$

The leakages under these conditions are determined by Eq. (D15) and (D27).

The eigenstate localization for strictly q-local perturbation in good cLDPC codes was already proved in [1]. Here, we follow a similar procedure to prove it, but use a slightly tighter bound obtained from our Theorem. 2. We remark that the method used in [1] is hard to extend to quasi-q-local perturbations, whereas our Theorem 2 can allow us to bound the leakage for quasi-q-local perturbations as well.

1. Eigenstate localization in cLDPC codes with strictly q-local perturbations

For the perturbed eigenstate $|\psi\rangle$ with energy $E' \equiv \varepsilon' n + E_g$, its energy difference from the top of W is $(b-\varepsilon')n$. Thus, we can choose the energy window to be $[E'-dn, E'+dn]$, with $d = b-\varepsilon'$. Note that here the b and ε' are defined in different eigenbases, i.e., the eigenbases of H_C and H, respectively. From Theorem 2, we know that the leakage outside all the clusters is bounded by (noting $P_> = 1 - P_W$)

$$\|P_> |\psi\rangle\| \leq \epsilon^{(2)}_{\lambda,\Delta_q}(b-\varepsilon'), \tag{D5}$$

which is exponentially small. Then we divide the perturbed eigenstate $|\psi\rangle$ in two parts

$$|\psi\rangle = |\eta\rangle + |\epsilon\rangle, \tag{D6}$$

where $|\eta\rangle \equiv P_W|\psi\rangle$ is in some clusters and $|\epsilon\rangle \equiv P_>|\psi\rangle$ is outside all clusters. Applying the projector P_W to $H|\psi\rangle = E'|\psi\rangle$ we have

$$P_W H P_W |\psi\rangle + P_W H(1-P_W)|\psi\rangle = E'|\eta\rangle. \tag{D7}$$

Defining the truncated Hamiltonian as $H_W \equiv P_W H P_W$, we obtain

$$(H_W - E')|\eta\rangle = P_W V_0 |\epsilon\rangle, \tag{D8}$$

where we have used $P_W H_C(1-P_W) = 0$ and $P_W H_{\mathrm{d}}(1-P_W) = 0$. Thus, $|\eta\rangle$ and E' are an approximate eigensolution of H_W, as $||\epsilon\rangle||$ is exponentially small. For simplicity, we denote the RHS norm after normalizing $|\eta\rangle$ as $\epsilon_> \equiv \frac{\|P_W V_0|\epsilon\rangle\|}{\sqrt{1-\||\epsilon\rangle\|^2}}$. Using $\|V_0\| \leq \lambda n$, we can bound $\epsilon_>$ by

$$\epsilon_> \leq 2\lambda n \epsilon^{(2)}_{\lambda,\Delta_q}(b-\varepsilon'), \tag{D9}$$

where we assume $\epsilon^{(2)}_{\lambda,\Delta_q}(b-\varepsilon') < 3/4$ so that $\frac{1}{\sqrt{1-\||\epsilon\rangle\|}} < 2$.

Note that H cannot directly connect different clusters w's due to the large distances between them, so $P_w H P_{w'} = 0$ for $w \neq w'$. Thus, H_W is automatically block diagonalized, with each block corresponding to each cluster w. Therefore, the eigenstates of H_W all reside within a single cluster w, and all P_w's commute with H_W. We can denote H_W's m-th eigenstate localized in cluster w as $|\phi_{w,m}\rangle$ with energy $E_{w,m}$. To show that the eigenstates of the whole Hamiltonian H also reside within a single cluster, we need an additional lemma.

Lemma D.1. For any Hamiltonian H, if one can find an approximate eigensolution $(H-E')|\Psi\rangle = |\epsilon\rangle$, where $|\Psi\rangle$ is normalized and $\||\epsilon\rangle\| = \epsilon < 1$, then there is at least one exact energy $E_\star$ of H satisfying

$$|E_\star - E'| \leq \epsilon. \tag{D10}$$

Let $\mathbb{V}$ be some subspace constructed from eigenstates of H that contains $|E_\star\rangle$. For any $|E_i\rangle \in \mathbb{V}$ and $|E_j\rangle \notin \mathbb{V}$,

we define $\delta_{\mathbb{V}} \equiv \min_{i,j} |E_j - E_i|$. If $\delta_{\mathbb{V}} > \epsilon$, then $|\Psi\rangle$ resides almost in $\mathbb{V}$, with leakage

$$\|(1 - P_{\mathbb{V}}) |\Psi\rangle \| \leq \frac{\epsilon}{\delta_{\mathbb{V}} - \epsilon}. \tag{D11}$$

We show the proof in Appendix D 4. Thus, there is at least one $|\phi_{w_\star, j_\star}\rangle$ whose energy is close to E'. By choosing $\mathbb{V} = w_\star$, we can obtain

$$\|(1 - P_{w_\star}) |\eta\rangle \| \leq \frac{\epsilon_>}{\delta_{w_\star} - \epsilon_>} \| |\eta\rangle \|, \tag{D12}$$

where $\delta_{w_\star}$ is the minimum spectral gap between states in cluster $w_\star$ and other clusters. In general, we can define

$$\delta_W \equiv \min_{w_1 \neq w_2} |E_{w_1,m_1} - E_{w_2,m_2}|. \tag{D13}$$

Since $\epsilon_>$ is exponentially small, as long as δ_W decays slower than $\epsilon_>$, the leakage of $|\eta\rangle$ outside the $w_\star$ can still be exponentially small. Therefore, we need the decaying speed of the gap between different clusters to be lower bounded, which can be stated as the following lemma:

Lemma D.2 (Limited level attraction between the clusters [1])**.** If the small diagonal detuning H_{d} is added, for any $0 < \xi_2 < \xi_1 - 2\ln 2$, we have

$$\mathbf{P}\left(\delta_W < e^{-\xi_1 n}\right) < e^{-\xi_2 n}. \tag{D14}$$

We show the proof in Appendix D 5. We can choose $\xi_1 = 2$ here for convenience. Thus, δ_W is at least e^{-2n} with high probability $1 - e^{-2(1-\ln 2)n}$.

Thus, with high probability $1 - e^{-2(1-\ln 2)n}$, the leakage outside $w_\star$ is bounded by

$$\|(1 - P_{w_\star}) |\psi\rangle \| \leq \exp\left(-\left(\xi - o(1)\right) n\right), \tag{D15}$$

where

$$\xi \equiv \frac{b - \varepsilon'}{\Delta_q} \ln \frac{b - \varepsilon'}{\lambda} - \frac{b - \varepsilon' - \lambda}{\Delta_q} - 2. \tag{D16}$$

We can verify that when

$$\lambda < \frac{b - \varepsilon'}{e} e^{-\frac{2\Delta_q}{b - \varepsilon'}} \equiv \lambda_{\mathrm{Eigen}}, \tag{D17}$$

we can ensure that $\xi > 0$.

2. Eigenstate localization in cLDPC codes with quasi-q-local perturbations

For a quasi-$q_\star$-local perturbation V_0, we can follow similar procedures. We consider an eigenstate $|\psi\rangle$ of

$$H = H_C + V_0 + H_{\mathrm{d}}, \tag{D18}$$

with energy $E' = \varepsilon' n + E_g$. From Theorem 2, we know that the leakage outside all the clusters is bounded by

$$\|P_> |\psi\rangle \| \leq \epsilon^{(1)}_{\lambda, \Delta_{q_\star}}(b - \varepsilon'), \tag{D19}$$

which is exponentially small. Then we divide the quasi-$q_\star$-local V_0 to the local part V_L and the remainder V_R, where V_L includes all terms in V_0 with k-locality satisfying $k < \nu_2$, and $V_R \equiv V_0 - V_L$. The local part V_L satisfies

$$P_{w'} V_L(t) P_w = 0, \text{ for } \; w \neq w', \tag{D20}$$

and the remainder V_R satisfies

$$\|V_R\| \leq \lambda n e^{-\nu_2/q_\star}. \tag{D21}$$

Similarly, we can define the truncated Hamiltonian H_W as

$$H_W \equiv P_W(H_C + V_L + H_{\mathrm{d}})P_W, \tag{D22}$$

and obtain

$$(H_W - E')\,|\eta\rangle = P_W V_0\,|\epsilon\rangle - P_W V_R P_W\,|\psi\rangle\,, \tag{D23}$$

where $|\eta\rangle \equiv P_W\,|\psi\rangle$ and $|\epsilon\rangle \equiv P_>\,|\psi\rangle$. We define

$$\epsilon_> \equiv \frac{\|P_W V_0\,|\epsilon\rangle - P_W V_R P_W\,|\psi\rangle\,\|}{\|\,|\eta\rangle\,\|}, \tag{D24}$$

As the norm of $|\epsilon\rangle$ is exponentially small, we assume it is less than 3/4, so that $\|\,|\eta\rangle\,\| \geq 1/2$. Then we can bound $\epsilon_>$ by

$$\epsilon_> \leq 2\lambda n \left(\epsilon^{(1)}_{\lambda,\Delta_{q_\star}}(b-\varepsilon') + e^{-\nu_2/q_\star}\right). \tag{D25}$$

Next, we want to use Lemma D.1 to bound the leakage outside a cluster w of $|\psi\rangle$. It is crucial to check whether the eigenstates of the truncated Hamiltonian H_W remain localized in each cluster. Although V_L now includes larger-k-local terms, k in V_L is strictly less than ν_2 by definition. Therefore, H_W still contains no interaction between different clusters. Each eigenstate of H_W remains within one single cluster w, and all P_w's commute with H_W. Thus, the Lemma D.1 still works here.

The next task is to bound δ_W, which can be defined similarly

$$\delta_W \equiv \min_{w_1 \neq w_2} |E_{w_1,m_1} - E_{w_2,m_2}|. \tag{D26}$$

One can verify that the Lemma D.2 also works as long as the eigenstates of H_W remain clustered in each w. Thus, We can choose $\xi_1 = 2$ here for convenience. Thus, δ_W is at least e^{-2n} with high probability $1 - e^{-2(1-\ln 2)n}$. Therefore, with high probability $1 - e^{-2(1-\ln 2)n}$, the leakage outside $w_\star$ is bounded by

$$\|(1 - P_{w_\star})\,|\psi\rangle\,\| \leq \exp\left(-\xi n\right), \tag{D27}$$

where

$$\xi \equiv \min\left(\frac{b-\varepsilon'-\lambda}{\Delta_{q_\star}} - \frac{\lambda}{\Delta_{q_\star}}\ln\frac{b-\varepsilon'}{\lambda}, \frac{\nu_2}{nq_\star}\right) - 2. \tag{D28}$$

We can choose $\lambda < \frac{b-\varepsilon'}{e}$ and adjust $q_\star$ to ensure that both terms are positive. This gives an upper bound for $q_\star$

$$\lambda < \frac{b-\varepsilon'}{e}, \quad q_\star \leq \min\left(\frac{(b-\varepsilon')(1-2/e)}{4M}, \frac{\nu_2}{2n}\right). \tag{D29}$$

3. Discussions on Eigenstate localization in qLDPC codes

To investigate the eigenstate localization in qLDPC codes, we can still use Theorem 2 to bound the $\|P_>\,|\psi\rangle\,\|$ exponentially.

However, an issue arises when we want to apply Lemma D.1 to bound the $\|(1-P_w)\,|\psi\rangle\,\|$, where we need to lower bound δ_W and ensure that it is much larger than the $\|P_>\,|\psi\rangle\,\|$. For strictly q-local V_0, we can upper bound $\|P_>\,|\psi\rangle\,\|$ by $\epsilon^{(2)}_{\lambda,\Delta_q}(b-\varepsilon')$. On the other hand, it is recently proved [13, 14] that the ground state degeneracy of good qLDPC codes can only be lifted with maximum energy difference bounded by $\lambda e^{-\Theta(n)}$ [14], which has similar scaling with $\epsilon^{(2)}_{\lambda,\Delta_q}$. In addition, this only gives an upper bound of δ_W but what we need is its lower bound. Since the upper bound is already exponentially small, it is unlikely to rigorously obtain a lower bound that is much larger than $\epsilon^{(2)}_{\lambda,\Delta_q}$.

While the ground state degeneracy cannot be lifted by local operators, we can consider detuning H_{d} including terms like

$$H_{\mathrm{d}} = \frac{1}{n^2}\sum_j h_j \bar{Z}_j, \tag{D30}$$

where $\bar{Z}_j$ is the logical Z operator for j-th logical qubit. Although such detuning term requires $O(n)$-body interaction and is unrealistic, we can consider it as a spontaneous symmetry breaking: H_{d} will be infinitesimal in the thermodynamic limit but is sufficient to lift all the degeneracies. Thus, although the eigenstate localization for qLDPC may not exist in finite systems, its dynamical stability can be understood from the SSB perspective.

4. Proof of Lemma D.1

Lemma D.1 For any Hamiltonian H, if one can find an approximate eigensolution $(H - E')\,|\Psi\rangle = |\epsilon\rangle$, where $|\Psi\rangle$ is normalized and $\|\,|\epsilon\rangle\,\| = \epsilon < 1$, then there is at least one exact energy $E_\star$ of H satisfying

$$|E_\star - E'| \leq \epsilon. \tag{D31}$$

Let $\mathbb{V}$ be some subspace constructed from eigenstates of H that contains $|E_\star\rangle$. Suppose that for any $|E_i\rangle \in \mathbb{V}$ and $|E_j\rangle \notin \mathbb{V}$, we define $\delta_{\mathbb{V}} \equiv \min_{i,j} |E_j - E_i|$. If $\delta_{\mathbb{V}} > \epsilon$, then $|\Psi\rangle$ resides almost in $\mathbb{V}$, with leakage

$$\|(1 - P_{\mathbb{V}})\,|\Psi\rangle\,\| \leq \frac{\epsilon}{\delta_{\mathbb{V}} - \epsilon}. \tag{D32}$$

Proof. We can expand $|\Psi\rangle$ in the eigenbasis of H,

$$|\Psi\rangle = \sum_j a_j\,|E_j\rangle\,. \tag{D33}$$

From $(H - E')\,|\Psi\rangle = |\epsilon\rangle$, we can obtain

$$|\epsilon\rangle = \sum_j a_j (E_j - E')\,|E_j\rangle \tag{D34}$$

Taking the norm square of both sides, we obtain

$$\epsilon^2 = \sum_j |a_j|^2 (E_j - E')^2 \tag{D35}$$

Since $\sum_j |a_j|^2 = 1$, there is at least one $E_\star$ in all E_j's satisfying

$$|E_\star - E'| \leq \epsilon, \tag{D36}$$

otherwise the RHS of Eq. (D35) is always greater than ϵ^2.

Then we can choose a subspace $\mathbb{V} \ni |E_\star\rangle$ and rewrite the expansion for $|\Psi\rangle$

$$|\Psi\rangle = \sum_j a_j^{\text{in}}\,|E_j^{\text{in}}\rangle + \sum_j a_j^{\text{out}}\,|E_j^{\text{out}}\rangle \tag{D37}$$

where $|E_j^{\text{in}}\rangle \in \mathbb{V}$ and $|E_j^{\text{out}}\rangle \notin \mathbb{V}$. Using $(H - E')\,|\Psi\rangle = |\epsilon\rangle$, we obtain

$$|\epsilon\rangle = \sum_j a_j^{\text{in}} (E_j^{\text{in}} - E')\,|E_j^{\text{in}}\rangle + \sum_j a_j^{\text{out}} (E_j^{\text{out}} - E')\,|E_j^{\text{out}}\rangle\,. \tag{D38}$$

By definition, we have $|E_j^{\text{out}} - E_\star| \geq \delta_{\mathbb{V}}$. With $|E_\star - E'| \leq \epsilon$, we can get $|E_j^{\text{out}} - E'| \geq \delta_{\mathbb{V}} - \epsilon$. Thus, we obtain

$$\epsilon^2 \geq \sum_j |a_j^{\text{out}}|^2 (E_j^{\text{out}} - E')^2 \geq (\delta_{\mathbb{V}} - \epsilon)^2 \sum_j |a_j^{\text{out}}|^2. \tag{D39}$$

Therefore, the leakage can be bounded by

$$\|(1 - P_{\mathbb{V}})\,|\Psi\rangle\,\| = \sqrt{\sum_j |a_j^{\text{out}}|^2} \leq \frac{\epsilon}{\delta_{\mathbb{V}} - \epsilon}. \tag{D40}$$

□

5. Proof of Lemma D.2

Lemma D.2. If the small diagonal detuning $H_{\rm d}$ is added

$$H_{\rm d} \equiv \frac{1}{n^2}\sum_j h_j^z Z_j, \tag{D41}$$

for any $0 < \xi_2 < \xi_1 - \ln 4$, we have

$$\mathbf{P}\left(\delta_W < e^{-\xi_1 n}\right) < e^{-\xi_2 n}. \tag{D42}$$

Here, δ_W is defined in the eigenbasis of

$$H_W \equiv P_W(H_C + V_0 + H_{\rm d})P_W, \tag{D43}$$

whose eigenstates are all exactly localized in a specific cluster w and can be labeled as $|\phi_{w,m}\rangle$ with energy $E_{w,m}$. Then we can define

$$\delta_W \equiv \min_{w_1 \neq w_2} |E_{w_1,m_1} - E_{w_2,m_2}|. \tag{D44}$$

Proof. For a specific cluster w with codeword $|{\rm w}\rangle$, we can write it as a bitstring ${\rm w} \equiv \{z_1^w, z_2^w ... z_n^w\}$, where each z_j^w is in $\{0,1\}$. Consider the following variable

$$h_j^w \equiv \frac{1}{\sqrt{n}}\sum_{k=1}^{n}(-1)^{z_k^w} e^{-i\frac{2\pi}{n}jk} h_k^z. \tag{D45}$$

One can find an orthogonal transformation to connect the random variables h_i^z to h_i^w, so that all h_i^w's also follow independent Gaussian distributions with zero mean and unit variance. The $H_{\rm d}$ become

$$H_{\rm d} = \frac{1}{n^{5/2}}\sum_{j=1}^{n} h_j^w \sum_{k=1}^{n} e^{i\frac{2\pi}{n}jk}(-1)^{z_k^w} Z_k. \tag{D46}$$

We can regard $H_W = H_C + V_0 + H_{\rm d}$ as a function of all $\{h_j^w\}$. Using the Hellmann-Feynman theorem, for arbitrary energy $E_{w,m}$ of H_W, we have

$$\frac{\partial E_{w,m}}{\partial h_j^w} = \left\langle \phi_{w,m} \left| \frac{\partial H_W}{\partial h_j^w} \right| \phi_{w,m} \right\rangle. \tag{D47}$$

Take $j = 1$ as an example, we obtain

$$\frac{\partial E_{w,m}}{\partial h_1^w} = \frac{1}{n^{5/2}}\left\langle \phi_{w,m} \left| \sum_{k=1}^{n}(-1)^{z_k^w} Z_k \right| \phi_{w,m} \right\rangle. \tag{D48}$$

Note that for a Z-basis state $|z\rangle$,

$$\left\langle z \left| \sum_{k=1}^{n}(-1)^{z_k^w} Z_k \right| z \right\rangle = n - 2\cdot \mathbf{D}({\rm w}, z), \tag{D49}$$

where $\mathbf{D}$ here is reduced to the Hamming distance. Since all Z-basis states inside w is at most γn far from w, we obtain

$$\frac{\partial E_{w,m}}{\partial h_1^w} \geq \frac{n - 2\gamma n}{n^{5/2}}. \tag{D50}$$

Similarly, for an arbitrary $w' \neq w$, all Z-basis states inside w' is at least $d_C - \gamma n$ far from w, we have

$$\begin{aligned}\frac{\partial E_{w',m'}}{\partial h_1^w} &= \frac{1}{n^{5/2}}\langle \phi_{w',m'}|\sum_{k=1}^{n}(-1)^{z_k^w} Z_k|\phi_{w',m'}\rangle \\ &\leq \frac{n - 2(d_C - \gamma n)}{n^{5/2}}.\end{aligned} \tag{D51}$$

Therefore, we obtain

$$\frac{\partial}{\partial h_1^w}(E_{w,m} - E_{w',m'}) \geq \frac{2(d_C - 2\gamma n)}{n^{5/2}} = \frac{2\nu_2}{n^{5/2}}. \tag{D52}$$

Now we can fix all the other h_j^w with $j \neq 1$ and regard $E_{w,m} - E_{w',m'}$ as a function of h_1^w, which increases monotonically. Thus, the interval length of h_1^w for $|E_{w,j} - E_{w',j'}| < e^{-\xi_1 n}$ is at most $\frac{n^{5/2}}{\nu_2} e^{-\xi_1 n}$. Taking into account all possible pairs for $E_{w,m}$ and $E_{w',m'}$, the total interval length is at most $4^n \frac{n^{5/2}}{\nu_2} e^{-\xi_1 n}$. Since h_1^w is chosen from a Gaussian with zero mean and unit variance, we have

$$\mathbf{P}(h_1^w) = \frac{1}{\sqrt{2\pi}} \exp\left(-\frac{(h_1^w)^2}{2}\right). \tag{D53}$$

Then the probability that h_1^w is within those intervals is bounded by

$$\mathbf{P}\left(\delta_W < e^{-\xi_1 n}\right) \leq \frac{n^{5/2}}{\nu_2 \sqrt{2\pi}} e^{-(\xi_1 - \ln 4)n} < e^{-\xi_2 n}, \tag{D54}$$

where we can choose ξ_2 in the range $0 < \xi_2 < \xi_1 - \ln 4$.

□

Appendix E: Proof of the slow mixing of Gibbs samplers

In this section, we investigate the efficiency of reaching thermal equilibrium of the c/qLDPC codes via a local Gibbs sampler, especially in low-temperature regions. A general Gibbs sampler can be any quantum channel $\mathcal{M}_H$ for a Hamiltonian H whose steady state is $\rho_0 = \frac{1}{\mathcal{Z}} e^{-\beta H}$, where $\mathcal{Z} \equiv \operatorname{tr} e^{-\beta H}$ is the partition function. In principle, by applying $\mathcal{M}$ on an arbitrary initial state t times, and if t is sufficiently large, the resultant density matrix can be arbitrarily close to the steady state of $\mathcal{M}$, which is the Gibbs state of H by definition. The minimal t required to reach sufficiently close to the steady state is called the mixing time.

We can always write $\mathcal{M}$ in Kraus operators

$$\mathcal{M}[\rho] = \sum_i K_i \rho K_i^\dagger. \tag{E1}$$

The locality constraint on $\mathcal{M}$ requires that all K_i's are strictly k-local where k is an $O(1)$ constant. Although we mainly focus on strictly k-local $\mathcal{M}$ here, the result can easily be extended to quasi-k-local channels [2].

We are going to prove that the Gibbs samplers for c/qLDPC codes are generally slow, even if (quasi-)q-local perturbations are added to H_C. In particular, we set $\mathcal{M}$ as a local Gibbs sampler for

$$H = H_C + V_0. \tag{E2}$$

We will show shortly that if an initial state is prepared in one cluster w, it takes an exponentially long time to converge to the steady state, which spreads in all clusters. The proof relies on the following quantum bottleneck theorem proved in [2].

Lemma E.1 (Quantum bottleneck theorem [2])**.** For a quantum channel $\mathcal{M}[\rho] = \sum_i K_i \rho K_i^\dagger$ whose steady state is ρ_0, suppose we can divide the Hilbert space into $\mathbb{H} = \mathbb{A} \oplus \mathbb{B}_1 \oplus \mathbb{B}_2 \oplus \mathbb{C}$, with corresponding projectors $P_{\mathbb{A},\mathbb{B}_1,\mathbb{B}_2,\mathbb{C}}$, and $\mathcal{M}$ satisfies

$$(P_{\mathbb{B}_2} + P_{\mathbb{C}})\, K_i P_{\mathbb{A}} = (P_{\mathbb{A}} + P_{\mathbb{B}_1})\, K_i P_{\mathbb{C}} = 0. \tag{E3}$$

Then we can find an approximate steady state $\rho_{\mathbb{A}} \equiv \frac{P_{\mathbb{A}} \rho_0 P_{\mathbb{A}}}{\operatorname{tr}(P_{\mathbb{A}} \rho_0)}$ with error bounded by

$$\|\mathcal{M}[\rho_{\mathbb{A}}] - \rho_{\mathbb{A}}\|_1 \leq 10 \frac{\sqrt{\operatorname{tr}(P_{\mathbb{B}} \rho_0)}}{\operatorname{tr}(P_{\mathbb{A}} \rho_0)} \equiv \epsilon_{\mathcal{M}}(\mathbb{A}), \tag{E4}$$

where $P_{\mathbb{B}} = P_{\mathbb{B}_1} + P_{\mathbb{B}_2}$.

From the Lemma, one can verify that if $\epsilon_{\mathcal{M}}(\mathbb{A})$ is exponentially small, an initial state localized in $\mathbb{A}$ will be stuck there for an exponentially long time.

Thus, the next step is to show that $\epsilon_{\mathcal{M}}(\mathbb{A})$ is indeed exponentially small even if (quasi-)q-local perturbations are added. The procedures for strictly q-local perturbations are similar to those in [2], except that we bound the high-energy contribution—the actual bottleneck of the Gibbs sampler $\mathcal{M}$—from Theorem. 2. In addition, we can also bound the bottleneck ratio for quasi-q-local perturbations from Theorem 2, which is beyond the methods in [2].

1. Strictly q-local perturbations

We can then choose $\mathbb{A} = w$, $\mathbb{C}$ being all the other clusters, and $\mathbb{B}$ being the high energy contribution with $\mathbb{B}_1$ contain all eigenstates of H_C that can be connected by local channel $\mathcal{M}$ with eigenstates in w. This gives

$$P_{\mathbb{A}} = P_w,\ P_{\mathbb{B}} = P_>,\ P_{\mathbb{C}} = \sum_{w' \neq w} P_{w'},\ \rho_0 = \frac{1}{\mathcal{Z}} e^{-\beta H}. \tag{E5}$$

where $\mathcal{Z} \equiv \operatorname{tr} e^{-\beta H}$ is the partition function. Thus, we obtain

$$\epsilon_{\mathcal{M}}(w) \leq 10\sqrt{\mathcal{Z}} \frac{\sqrt{\operatorname{tr}\left(P_> e^{-\beta H}\right)}}{\operatorname{tr}\left(P_w e^{-\beta H}\right)}. \tag{E6}$$

Note that we can always upper bound $\mathcal{Z}$ by

$$\mathcal{Z} \leq 2^n e^{-\beta(E_g - \lambda n)}, \tag{E7}$$

where E_g is the ground state energy of H_C and $E_g - \lambda n$ is the lowest possible ground state energy of H via Weyl's inequality. The lower bound of $\operatorname{tr}(P_w \rho_0)$ can be obtained by using $P_w = \sum_{|\psi\rangle \in w} |\psi\rangle\langle\psi|$, so that

$$\begin{aligned} \operatorname{tr}\left(P_w e^{-\beta H}\right) &\geq \max_{|\psi\rangle \in w} \operatorname{tr}(e^{-\beta H} |\psi\rangle\langle\psi|) \\ &\geq e^{-\beta(E_g + \lambda n)}. \end{aligned} \tag{E8}$$

In the second line, we have used the fact that any state in the cluster w has an energy $E \leq E_g + \lambda n$. The upper bound of $\operatorname{tr}\left(P_> e^{-\beta H}\right)$ can be obtained by the following inequality, which is defined for an arbitrary projector P to a subspace S

$$\operatorname{tr}(PA) = \operatorname{tr}(PAP) \leq \operatorname{rank}(P) \cdot \lambda_{\max}(PAP) = \operatorname{rank}(P) \cdot \max_{\substack{u \in S \\ \|u\|=1}} \langle u|A|u\rangle \leq \operatorname{rank}(P) \cdot \max_{i, P|\phi_i\rangle \neq 0} \langle \phi_i|A|\phi_i\rangle, \tag{E9}$$

where A can be any Hermitian operators with all eigenstates denoted by $|\phi_i\rangle$, and we use $\lambda_{\max}(A)$ (different from the notation λ for perturbation strength) to denote the maximum eigenvalue of a Hermitian operator A. The last step can be easily proved by expanding arbitrary subspace state u in the eigenbasis of A. Similarly, if we denote $|\phi_i\rangle$ as eigenstates of perturbed Hamiltonian H

$$\operatorname{tr}\left(P_> e^{-\beta H}\right) \leq 2^n \max_{i, P_>|\phi_i\rangle \neq 0} \left\langle \phi_i \,\middle|\, e^{-\beta H} \,\middle|\, \phi_i \right\rangle. \tag{E10}$$

From Theorem 2, we can bound the expectation by expanding $|\phi_i\rangle$ in the eigenbasis of H, where we swap the roles of H and H_C. Suppose the energy of $|\phi\rangle$ in H is $E_\phi \geq E_g + bn$, we know that $|\phi\rangle$ is exponentially localized in the energy window $\mathcal{E}_\phi \equiv [E_\phi - dn, E_\phi + dn]$ of H, where d can be any constant larger than λ. By setting $|\phi\rangle = \sum_j a_j |\psi_j\rangle$ and $|\psi_j\rangle$ is an eigenstate of H with energy E_j, we have the following for any $|\phi\rangle \in \{|\phi_i\rangle\}$.

$$\langle\phi|e^{-\beta H}|\phi\rangle = \sum_{j, E_j \in \mathcal{E}_\phi} |a_j|^2 e^{-\beta E_j} + \sum_{j, E_j \notin \mathcal{E}_\phi} |a_j|^2 e^{-\beta E_j} \leq e^{-\beta(E_\phi - dn)} + e^{-\beta E_g} \epsilon^{(2)}_{\lambda, \Delta_q}(d). \tag{E11}$$

Thus, we have

$$\operatorname{tr}\left(P_> e^{-\beta H}\right) \leq e^{n \ln 2 - \beta E_g} \left(e^{-\beta(b-d)n} + \epsilon^{(2)}_{\lambda, \Delta_q}(d)\right). \tag{E12}$$

Then we can bound $\epsilon_{\mathcal{M}}(w)$ by

$$\frac{\epsilon_{\mathcal{M}}(w)^2}{100} \leq e^{n(2\ln 2+3\beta\lambda)}\left(e^{-\beta(b-d)n}+\epsilon^{(2)}_{\lambda,\Delta_q}(d)\right). \tag{E13}$$

For convenience, we choose $d=b/4$, we have

$$\epsilon_{\mathcal{M}}(w)\leq e^{-\xi n}, \tag{E14}$$

where

$$\xi \equiv \min\left(\frac{3\beta(b-4\lambda)}{8}, \frac{\frac{b}{4}\ln\frac{b}{4e\lambda}+\lambda}{2\Delta_q}-\frac{3}{2}\beta\lambda\right)-\ln 2. \tag{E15}$$

To ensure that $\xi>0$, we require that

$$\beta>\frac{8\ln 2}{3b},\ \lambda<\min\left(\frac{b}{4e}e^{-\Delta_q(3\beta+\frac{8\ln 2}{b})}, \frac{3\beta b}{4}-2\ln 2\right). \tag{E16}$$

2. Quasi-q-local perturbations

One can verify that for Quasi-$q_\star$-local perturbation V_0, most of the steps are similar to strictly q-local cases, except upper bounding $\mathrm{tr}(P_>e^{-\beta H})$. This can be similarly bounded by

$$\mathrm{tr}\left(P_>e^{-\beta H}\right)\leq 2^n \max_{P_>|\phi\rangle\neq 0}\left\langle\phi\right| e^{-\beta H}\left|\phi\right\rangle. \tag{E17}$$

From Theorem 2 and Eq. (E11) we know that

$$\langle\phi|e^{-\beta H}|\phi\rangle \leq e^{-\beta(E_\phi-dn)}+e^{-\beta E_g}\epsilon^{(1)}_{\lambda,\Delta_{q_\star}}(d), \tag{E18}$$

where the only difference is replacing $\epsilon^{(2)}_{\lambda,\Delta_q}$ with $\epsilon^{(1)}_{\lambda,\Delta_{q_\star}}$. Similarly, we have

$$\frac{\epsilon_{\mathcal{M}}(w)^2}{100} \leq e^{n(2\ln 2+3\beta\lambda)}\left(e^{-\beta(b-d)n}+\epsilon^{(1)}_{\lambda,\Delta_{q_\star}}(d)\right). \tag{E19}$$

For convenience, we choose $d=b/4$, we have

$$\epsilon_{\mathcal{M}}(w)\leq e^{-\xi n}, \tag{E20}$$

where

$$\xi \equiv \min\left(\frac{3\beta(b-4\lambda)}{8}, \frac{\frac{b}{4}-\lambda\ln\frac{be}{4\lambda}}{2\Delta_{q_\star}}-\frac{3}{2}\beta\lambda\right)-\ln 2. \tag{E21}$$

To ensure that $\xi>0$, we can choose the following requirements:

$$\beta>\frac{8\ln 2}{3b},\ \lambda<\min\left(\frac{b}{4e}, \frac{3\beta b}{4}-2\ln 2\right),\ q_\star<\frac{b}{2M}\cdot\frac{e-2}{2e\ln 2+3\beta b}. \tag{E22}$$

Appendix F: Some bounds for nested commutators

In this section, we provide some useful bounds for the nested commutator $\mathrm{ad}^m_H(V)$ along with their asymptotic behavior under different conditions.

1. H is p-local and V is q-local

We consider a p-local H and a q-local V. They can be written in terms supported on different sites:

$$H = \sum_A h_A, \ V = \sum_B v_B, \tag{F1}$$

where each support contains at most p sites for A and at most q for B. In addition, we require H to satisfy

$$\sum_{A \ni i} \|h_A\| \leq M \tag{F2}$$

for any site i. We first consider the case where $q < p$. Define $\Delta_{p-1} \equiv 2(p-1)M$, the norm of the nested commutators are bounded by

$$\|\mathrm{ad}_H^m(V)\| \leq m! \Delta_{p-1}^m \|V\|_{\mathrm{X}}, \tag{F3}$$

where $\|V\|_{\mathrm{X}} \equiv \sum_X \|v_X\|$. We do this by induction. For $m = 0$ the inequality is trivial. For $m = 1$, we have

$$\mathrm{ad}_H(V) = \sum_B \sum_{A, A\cap B \neq \emptyset} [h_A, v_B]. \tag{F4}$$

For any single term v_B on the support B, it contains q sites and $q \leq p-1$, so

$$\begin{aligned} \left\| \sum_{A, A\cap B\neq\emptyset} [h_A, v_B] \right\| &\leq \sum_{i, i\in B} \sum_{A, A\ni i} \|[h_A, v_B]\| \\ &\leq \sum_{i, i\in B} 2M\|v_B\| \leq 2(p-1)M\|v_B\| = \Delta_{p-1}\|v_B\|, \end{aligned} \tag{F5}$$

Thus,

$$\|\mathrm{ad}_H(V)\| \leq \sum_B \Delta_{p-1}\|v_B\| = \Delta_{p-1}\|V\|_{\mathrm{X}}. \tag{F6}$$

Now we consider $m = 2$. Note that we can still write $\mathrm{ad}_H(V)$ as

$$\mathrm{ad}_H(V) = \sum_B v_B^{(1)}, \tag{F7}$$

where

$$v_{B^{(1)}}^{(1)} \equiv v_B^{(1)} = \sum_{A, A\cap B\neq\emptyset} [h_A, v_B]. \tag{F8}$$

We use $v_{B^{(1)}}^{(1)}$ to replace the notion of $v_B^{(1)}$ because it is now supported on $B^{(1)}$, which is an extension of original B and contains at most $2(p-1)$ sites (since H is p-local). By the above analysis, we know that the norm of $v_{B^{(1)}}^{(1)}$ is bounded by $\Delta_{p-1}\|v_B\|$. Thus,

$$\mathrm{ad}_H^2(V) = \sum_{B^{(1)}} \sum_{A, A\cap B^{(1)}\neq\emptyset} [h_A, v_{B^{(1)}}^{(1)}]. \tag{F9}$$

By similar procedure, we obtain

$$\|\mathrm{ad}_H^2(V)\| \leq 4(p-1)M\Delta_{p-1}\|V\|_{\mathrm{X}} = 2\Delta_{p-1}^2\|V\|_{\mathrm{X}}. \tag{F10}$$

Since $B^{(k)}$ grows with at most $(p-1)$ sites from $B^{(k-1)}$, $B^{(k)}$ is supported on at most $k(p-1)$ sites. Then the factor multiplied at k-th step is $2k(p-1)M = k\Delta_{p-1}$. Thus, by induction we have

$$\|\mathrm{ad}_H^m(V)\| \leq m! \Delta_{p-1}^m \|V\|_{\mathrm{X}}. \tag{F11}$$

For $q \geq p$, by the similar procedure as above we can obtain the bound

$$\begin{aligned} \|\mathrm{ad}_H^m(V)\| &\leq \frac{\Gamma(\frac{q}{p-1}+m)}{\Gamma(\frac{q}{p-1})}\Delta_{p-1}^m \|V\|_{\mathrm{X}} \\ &< m!\Delta_q^m \|V\|_{\mathrm{X}} . \end{aligned} \tag{F12}$$

Thus, if $H+V$ is k-local, we can always have

$$\|\mathrm{ad}_H^m(V)\| < m!\Delta_k^m \|V\|_{\mathrm{X}} . \tag{F13}$$

2. H is mutually commuting and V is q-local

Here we assume that the Hamiltonian H can be decomposed as

$$H = \sum_X h_X, \tag{F14}$$

where each h_X is supported on X and commutes with each other but can couple arbitrarily many sites, as long as

$$\sum_{X, X\ni i} \|h_X\| \leq M. \tag{F15}$$

For $m=1$, we have

$$\mathrm{ad}_H(V) = \sum_B \sum_{A, A\cap B\neq\emptyset} [h_A, v_B]. \tag{F16}$$

We can obtain the following

$$\begin{aligned} \left\| \sum_{A, A\cap B\neq\emptyset} [h_A, v_B] \right\| &\leq \sum_{i, i\in B} \sum_{A, A\ni i} \|[h_A, v_B]\| \\ &\leq \sum_{i, i\in B} 2M\|v_B\| = 2qM\|v_B\| = \Delta_q \|v_B\|, \end{aligned} \tag{F17}$$

where we define $\Delta_q \equiv 2qM$. For $m=2$, we can still write $\mathrm{ad}_H(V)$ as

$$\mathrm{ad}_H(V) = \sum_B v_B^{(1)}, \tag{F18}$$

where

$$v_B^{(1)} = \sum_{A, A\cap B\neq\emptyset} [h_A, v_B]. \tag{F19}$$

Now we consider when $[H_C, v_B^{(1)}]$ can be non-zero. If $C\cap B=\emptyset$, then $[H_C, v_B]=0$. By definition, we also have $[H_C, h_A]=0$ for arbitrary A. So $[H_C, v_B^{(1)}]=0$ if $C\cap B=\emptyset$. Thus, although the support of $v_B^{(1)}$ expands from v_B, $[H_C, v_B^{(1)}]$ is nonzero only if $C\cap B\neq\emptyset$. Thus, we can obtain

$$\mathrm{ad}_H^2(V) = \sum_B \sum_{C, C\cap B\neq\emptyset} [H_C, v_B^{(1)}], \tag{F20}$$

and thus bound the terms for each initial support B by

$$\begin{aligned} \left\| \sum_{C, C\cap B\neq\emptyset} [H_C, v_B^{(1)}] \right\| &\leq \sum_{i, i\in B} \sum_{C, C\ni i} \left\| [H_C, v_B^{(1)}] \right\| \\ &\leq \sum_{i, i\in B} 2M\|v_B^{(1)}\| = 2qM\|v_B^{(1)}\| \leq \Delta_q^2 \|v_B\|. \end{aligned} \tag{F21}$$

Thus, by induction we have

$$\|\mathrm{ad}_H^m(V)\| \leq \Delta_q^m \|V\|_{\mathrm{X}} . \tag{F22}$$

3. H is mutually commuting and V is quasi-q-local

By the definition of quasi-q-local $V = \sum_B v_B$ in Appendix A, we have

$$\sum_{B,|B|=k} \|v_B\| \le \frac{\|V\|_{q\text{-X}}}{q+1} e^{-k/q}. \tag{F23}$$

where the additional factor $\frac{1}{q+1}$ ensures that the total termwise norm $\|V\|_{\mathrm{X}}$ will be bounded by $\|V\|_{q\text{-X}}$ regardless of q. Using Eq. (F13), we have the following bounds

$$\begin{aligned} \|\mathrm{ad}_H^m(V)\| &\le (2M)^m \sum_{k=1}^n k^m \sum_{B,|B|=k} \|v_B\| \\ &\le \|V\|_{q\text{-X}} \frac{(2M)^m}{q+1} \sum_{k=1}^n k^m e^{-k/q}. \end{aligned} \tag{F24}$$

Here we give a rough but simple estimation to bound the summation at RHS by $m!$. Note that $x^m e^{-x/q}$ is a unimodal function, and for a unimodal function $f(x)$ we have $\sum_{x=a}^b f(x) \le \int_a^b f(x)\mathrm{d}x + \max_{x\in[a,b]} f(x)$. Then we have

$$\begin{aligned} \sum_{k=1}^\infty k^m e^{-k/q} &\le \int_0^\infty x^m e^{-x/q}\mathrm{d}x + \max_k k^m e^{-k/q} \\ &\le q^{m+1} m! + (mq/e)^m \\ &= (q+1)q^m m!\left(\frac{q}{q+1} + \frac{1}{q+1}\frac{m^m}{e^m m!}\right). \end{aligned} \tag{F25}$$

One can verify that $\frac{m^m}{e^m m!}$ is strictly decreasing for integer $m \ge 1$, so $\frac{m^m}{e^m m!} \le \frac{1}{e}$, Then we have

$$\begin{aligned} \frac{1}{q+1} \sum_{k=1}^\infty k^m e^{-k/q} &\le q^m m! \frac{q+1/e}{q+1} \\ &< q^m m! \end{aligned} \tag{F26}$$

Thus, we have

$$\|\mathrm{ad}_H^m(V)\| < \Delta_q^m m! \|V\|_{q\text{-X}}. \tag{F27}$$

[1] C. Yin, R. Nandkishore, and A. Lucas, Eigenstate localization in a many-body quantum system, Physical Review Letters **133**, 137101 (2024).

[2] T. Rakovszky, B. Placke, N. P. Breuckmann, and V. Khemani, Bottlenecks in quantum channels and finite temperature phases of matter, arXiv preprint arXiv:2412.09598 (2024).

[3] D. Gamarnik, B. T. Kiani, and A. Zlokapa, Slow mixing of quantum gibbs samplers, arXiv preprint arXiv:2411.04300 (2024).

[4] D. E. Parker, X. Cao, A. Avdoshkin, T. Scaffidi, and E. Altman, A universal operator growth hypothesis, Phys. Rev. X **9**, 041017 (2019).

[5] A. Weisse, R. Gerstner, and J. Sirker, Operator growth in disordered spin chains: Indications for the absence of many-body localization, Phys. Rev. Res. **7**, 033018 (2025).

[6] T. Kuwahara, T. Mori, and K. Saito, Floquet–magnus theory and generic transient dynamics in periodically driven many-body quantum systems, Annals of Physics **367**, 96 (2016).

[7] Z. Gong and R. Hamazaki, Bounds in nonequilibrium quantum dynamics, International Journal of Modern Physics B **36**, 2230007 (2022).

[8] D. A. Abanin, W. De Roeck, W. W. Ho, and F. Huveneers, Effective hamiltonians, prethermalization, and slow energy absorption in periodically driven many-body systems, Physical Review B **95**, 014112 (2017).

[9] D. Abanin, W. De Roeck, W. W. Ho, and F. Huveneers, A rigorous theory of many-body prethermalization for periodically driven and closed quantum systems, Communications in Mathematical Physics **354**, 809 (2017).

[10] D. V. Else, B. Bauer, and C. Nayak, Prethermal phases of matter protected by time-translation symmetry, Physical Review X **7**, 011026 (2017).

[11] J. Riordan, Moment recurrence relations for binomial, poisson and hypergeometric frequency distributions, The Annals of Mathematical Statistics **8**, 103 (1937).

[12] T. K. Philips and R. Nelson, The moment bound is tighter than chernoff's bound for positive tail probabilities, The American Statistician **49**, 175 (1995).

[13] W. De Roeck, V. Khemani, Y. Li, N. O'Dea, and T. Rakovszky, Low-density parity-check stabilizer codes as gapped quantum phases: Stability under graph-local perturbations, PRX Quantum **6**, 030330 (2025).

[14] C. Yin and A. Lucas, Low-density parity-check codes as stable phases of quantum matter, PRX Quantum **6**, 030329 (2025).
