# Supplementary material for: Universal energy-space localization and stable quantum phases against time-dependent perturbations
Source: Nat Commun. 2026 Jul 14;17:8643. doi: 10.1038/s41467-026-75465-z (PMC13486644; doi:10.1038/s41467-026-75465-z)
Supplement: Supplementary file 2 [file 41467_2026_75465_MOESM2_ESM.pdf]

File Name: Supplementary Data 1
Description: The file contains Mathematica codes for generating and plotting the data to produce Fig. 1.
