# Supplementary material for: Universal energy-space localization and stable quantum phases against time-dependent perturbations
Source: Nat Commun. 2026 Jul 14;17:8643. doi: 10.1038/s41467-026-75465-z (PMC13486644; doi:10.1038/s41467-026-75465-z)
Supplement: Supplementary file 3 [file 41467_2026_75465_MOESM4_ESM.pdf]

nature portfolio

Peer Review File

Universal energy-space localization and stable quantum phases against time-dependent perturbations

Corresponding Author: Mr Hongye Yu

This manuscript has been previously reviewed at another journal. This document only contains information relating to versions considered at Nature Communications.

This file contains all reviewer reports in order by version, followed by all author rebuttals in order by version.

Attachments originally included by the reviewers as part of their assessment can be found at the end of this file.

Version 0:

Reviewer comments:

Reviewer #1

(Remarks to the Author)

Open Access This Peer Review File is licensed under a Creative Commons Attribution 4.0 International License, which permits use, sharing, adaptation, distribution and reproduction in any medium or format, as long as you give appropriate credit to the original author(s) and the source, provide a link to the Creative Commons license, and indicate if changes were made.
In cases where reviewers are anonymous, credit should be given to 'Anonymous Referee' and the source.
The images or other third party material in this Peer Review File are included in the article's Creative Commons license, unless indicated otherwise in a credit line to the material. If material is not included in the article's Creative Commons

license and your intended use is not permitted by statutory regulation or exceeds the permitted use, you will need to obtain permission directly from the copyright holder.
To view a copy of this license, visit https://creativecommons.org/licenses/by/4.0/

Response to reviewers

Response to Reviewer 1

[We note reviewers' comments are shown in blue and our responses are in black.]
I appreciate the authors' efforts to reply to my comments. For the most part I think the manuscript is improved. As long as the authors agree with my understanding below, then I am not concerned about Appendix C anymore (and I had a partial misunderstanding when I read the paper before). My current understanding is that the authors cannot rule out the scenario of de-localization if I have, e.g., a confining LDPC H_C and a $\epsilon V(t)$ where $V(t)$ fluctuates (although is always a small perturbation) such that $\int \epsilon \|V'(t)\| \mathrm{d}t \gg n$, with $\|H_C\| \sim n$. This was what my last comment was concerned about, but if the authors are only addressing the case $\int \epsilon \|V'(t)\| \mathrm{d}t \sim \lambda n \ll n$ then indeed there is no problem.
Our response: We thank and agree with the reviewer for this clarification that our theorem only works for $\int \epsilon \|V'(t)\| \mathrm{d}t \sim \lambda n$, where λn should be smaller than the energy barrier but λ can still be $\Theta(1)$.
The reason this issue is very important to be unambiguous about is that in the vast majority of problems involving adiabatic optimization, one does not have the parameter λ being small. I think the added text in Section IV of the main text did help to explain this issue to the reader, although it might be nice to more explicitly state that the challenge with fully disproving the adiabatic algorithm is that λ is not generally small. The authors may wish to emphasize this point around where they discuss ruling out all polynomial time algorithms – I assume what one thing they refer to here as a potential loophole is choosing to wiggle $V(t)$ in such a way that λ is very large and their theorem does not apply. I agree that their theorem does not cover this case and I was concerned in my previous review that some of their lemmas claimed to address this situation.
Our response: We thank the reviewer for this comment and we have added a few sentences in the "Localization as an algorithmic barrier in hard optimization problems" subsection (previous Section IV) to address the reviewer's comment on adiabatic optimization that the majority (when $s(t)$ is away from 1 by a small constant depending on the energy barrier) of the evolution does not have small total variation (λn) so that our theorem does not apply, supplementing our previous description that our theorem only apply to the evolution near the end of the adiabatic evolution where the corresponding λ is below the barrier.

I appreciate the authors' efforts to reply to my comments. For the most part I think the manuscript is improved. As long as the authors agree with my understanding below, then I am not concerned about Appendix C anymore (and I had a partial misunderstanding when I read the paper before). My current understanding is that the authors cannot rule out the scenario of de-localization if I have, e.g., a confining LDPC H_C and a $\epsilon V(t)$ where $V(t)$ fluctuates (although is always a small perturbation) such that $\int \epsilon \|\dot{V}(t)\| \mathrm{d}t \gg n$, with $\|H_C\| \sim n$. This was what my last comment was concerned about, but if the authors are only addressing the case $\int \epsilon \|\dot{V}(t)\| \mathrm{d}t \sim \lambda n \ll n$ then indeed there is no problem.

The reason this issue is very important to be unambiguous about is that in the vast majority of problems involving adiabatic optimization, one does not have the parameter λ being small. I think the added text in Section IV of the main text did help to explain this issue to the reader, although it might be nice to more explicitly state that the challenge with fully disproving the adiabatic algorithm is that λ is not generally small. The authors may wish to emphasize this point around where they discuss ruling out all polynomial time algorithms – I assume what one thing they refer to here as a potential loophole is choosing to wiggle $V(t)$ in such a way that λ is very large and their theorem does not apply. I agree that their theorem does not cover this case and I was concerned in my previous review that some of their lemmas claimed to address this situation.
